# Analysis of the corporate political activity of major food industry actors in Fiji

**DOI:** 10.1186/s12992-016-0158-8

**Published:** 2016-05-10

**Authors:** Melissa Mialon, Boyd Swinburn, Jillian Wate, Isimeli Tukana, Gary Sacks

**Affiliations:** World Health Organization Collaborating Centre for Obesity Prevention, Deakin University, 221 Burwood Highway, Burwood, 3125 VIC Australia; School of Population Health, University of Auckland, Auckland, New Zealand; Pacific Research Center for the Prevention of Obesity and Non-Communicable Diseases (C-POND), College of Medicine, Nursing and Health Sciences, Fiji National University, Suva, Fiji; Ministry of Health, Suva, Fiji

**Keywords:** Policy, Food industry, Corporate political activity, Non-communicable diseases

## Abstract

**Background:**

Non-communicable diseases (NCDs) are the leading cause of mortality in Fiji, a middle-income country in the Pacific. Some food products processed sold and marketed by the food industry are major contributors to the NCD epidemic, and the food industry is widely identified as having strong economic and political power. However, little research has been undertaken on the attempts by the food industry to influence public health-related policies and programs in its favour. The “corporate political activity” (CPA) of the food industry includes six strategies (information and messaging; financial incentives; constituency building; legal strategies; policy substitution; opposition fragmentation and destabilisation). For this study, we aimed to gain a detailed understanding of the CPA strategies and practices of major food industry actors in Fiji, interpreted through a public health lens.

**Methods and results:**

We implemented a systematic approach to monitor the CPA of the food industry in Fiji for three months. It consisted of document analysis of relevant publicly available information. In parallel, we conducted semi-structured interviews with 10 stakeholders involved in diet- and/or public health-related issues in Fiji. Both components of the study were thematically analysed. We found evidence that the food industry adopted a diverse range of strategies in an attempt to influence public policy in Fiji, with all six CPA strategies identified. Participants identified that there is a substantial risk that the widespread CPA of the food industry could undermine efforts to address NCDs in Fiji.

**Conclusions:**

Despite limited public disclosure of information, such as data related to food industry donations to political parties and lobbying, we were able to identify many CPA practices used by the food industry in Fiji. Greater transparency from the food industry and the government would help strengthen efforts to increase their accountability and support NCD prevention. In other low- and middle-income countries, it is likely that a systematic document analysis approach would also need to be supplemented with key informant interviews to gain insight into this important influence on NCD prevention.

**Electronic supplementary material:**

The online version of this article (doi:10.1186/s12992-016-0158-8) contains supplementary material, which is available to authorized users.

## Background

Non-communicable diseases (NCDs) are the leading cause of mortality globally [1]. In Fiji, a middle income country (MIC) in the Pacific region [2], 80 % of all deaths can be attributed to NCDs [3]. In the Fijian population, poor diets, coupled with high rates of obesity (a third of Fijians were obese in 2008), are major risk factors for developing NCDs [4, 5]. The increased supply and marketing of unhealthy food products has been identified as one of the principal drivers of these risks factors [6–9]. Recent evidence suggests that the development and implementation of policies that could address obesity and NCDs has been slow and insufficient in Fiji, due to a range of factors including: the way the issue has been framed, concerns about potential impacts on economic development, low food self-sufficiency, limited evidence of policy effectiveness, and limited cooperation between different actors [10]. There is also increasing recognition, globally and in Fiji, of the attempts by major food industry actors to influence public policies in their favour [10, 11]. This “corporate political activity” (CPA) has the potential to compromise public health-related policies and programs because the commercial objective to maximise company profits and return to shareholders is potentially at odds with the objective of improving population health [11]. The CPA of the food industry has been described as consisting of six complementary strategies, including: information and messaging; financial incentives; constituency building; legal strategies; policy substitution; opposition fragmentation and destabilisation [11] (Refer to Additional file 1 for a detailed description of CPA strategies). While a recent study mentioned aspects of the CPA of the food industry, including the way the food industry has promoted de-regulation and established relationships with the media [10], the practices of the food industry in this country and their impact on policy have not been comprehensively analysed.

In light of the lack of implementation of recommended policies, and the perceived influence of the food industry on public health-related policies, public health advocates have called for greater transparency and accountability from governments and the food industry in this area [1, 12–14]. A detailed understanding of the CPA of the food industry has the potential to promote transparency and strengthen accountability mechanisms [12]. The International Network for Food and Obesity/NCDs Research, Monitoring and Action Support (INFORMAS) aims to monitor key aspects of food environments related to obesity and NCDs, including the policies and actions of the food industry [15]. As part of INFORMAS, we have proposed a systematic approach, adapted from approaches for monitoring the CPA of the tobacco industry using publicly-available information, to identify and monitor the CPA of the food industry with respect to public health [11]. In this study, we implemented this systematic approach in Fiji, supplemented by interviews with key informants, with the aim of identifying and understanding of the CPA strategies and practices of major food industry actors in Fiji, from a public health perspective.

## Methods

For this study, we took a critical stance, where the political influence of the food industry was considered as a potential determinant of ill health. The critical approach informed the way we collected, analysed and reported data.

This project was approved by the Fiji Research and Ethics Council from the Ministry of Education, Heritage and Arts (project number RA 11/15), the Fiji National Health Research Ethics Review Committee (project number 2014.104.MC) and the Human Ethics Advisory Groups of the Faculty of Health at Deakin University, Australia (project number HEAG-H 145_2014).

We carried out this study for a period of three months, between March and May 2015. For annual or occasional events, such as submissions to public consultations, elections, or conferences, we included the most recent data available (up to two years retrospectively), as detailed in Additional file 2.

For the first component of the study, we systematically collected and analysed information from a range of publicly-available sources in an effort to identify and monitor the CPA of the food industry with respect to public health [11]. The sources of information we targeted included food industry own materials, government materials, as well as other materials, such as the media (Additional file 2) [11]. Importantly, a number of sources of information that were recommended for systematically monitoring the CPA of the food industry [11] were either not available or available only to a limited extent at the time of data collection in Fiji (see Table 1). All information we retrieved was in English only.

**Table 1:** Availability of sources of information recommended to monitor the CPA of the food industry during the period of data collection (March to May 2015) in Fiji

Available sources of information	Unavailable sources of information
• Country-specific website for each industry actor • Industry actor social media accounts (Facebook, but no Twitter accounts) • News and media releases from the Fijian government • News articles related to the selected food industry actors and diet-related issues in major national newspapers	• From the Parliament and for the Ministries (and related agencies) responsible for diet- and public health-related issues: ○ Declarations of interests of all members ○ Submissions to public consultations from the food industry on diet- and public health-related issues ○ List of working groups on diet-related issues and the conflicts of interest of their members ○ List of public-private initiatives on diet-related issues ○ Minister’s diary disclosures ○ Register of lobbyists ○ Formal Freedom of Information (FOI) requests, or equivalent legislation through which researchers could have asked for: ▪ A list of meetings of food industry representatives from the selected sample of industry actors, with officials and/or representatives of the government ▪ Minutes and other reports of these meetings ▪ All correspondence (including emails) between food industry representatives from the selected sample of industry actors and officials and/or representatives from the government • Donations for elections from the food industry • Political parties annual returns for donations from the food industry • For major universities with a school/department of nutrition/dietetics or physical activity: ○ Research projects, fellowships or grants funded by the selected food industry actors ○ Prizes or awards offered to students by selected food industry actors

We collected data for 14 key food industry actors in Fiji, as listed in Table 2. We selected these food industry actors based on a previous identification of key food industry actors for Fiji [16], and on the availability (in March 2015) of at least a national website or a Facebook page/Twitter account for each actor, as suggested for the systematic monitoring of CPA strategies [11]. In addition, where we identified data related to other food companies, this was also noted. For example, we did not monitor systematically the activities of the company Nestle per se, given that it does not have a national website or Facebook page in Fiji, but we may have found indirectly data related to this company through other sources of information.

**Table 2:** List of selected food industry actors and corresponding websites (consulted between March and May 2015)

List of food industry actors selected for the document analysis	Core activity in Fiji	Corresponding websites
McDonald's	Fast food restaurant	http://www.mcdonaldsfiji.com/
Pizza King	Fast food restaurant	http://www.pizzaking.com.fj/about-us.html
Coca Cola Amatil	Sweetened beverages manufacturer/supplier	http://www.ccamatilfiji.com/
Tappoo	Sweetened beverages manufacturer/supplier	http://www.tappoo.com.fj/
Motibhai	Sweetened beverages manufacturer/supplier	http://www.motibhai.com/
Frezsco	Sweetened beverages manufacturer/supplier	http://www.frezcofiji.com/
Pinto	Sweetened beverages manufacturer/supplier	http://pintoindustries.com.fj/index.html
FMF	Processed food products manufacturer	http://www.fmf.com.fj/ https://www.facebook.com/FMFFoods
Foods Pacific	Processed food products manufacturer	http://foodspacific.com/ https://www.facebook.com/foodspacificltd
C J Patel	Processed food products manufacturer	Southern Cross Foods (subsidiary Fiji) http://www.scfoods.com.fj/
Food processors	Processed food products manufacturer	http://www.foodprocessors.com.fj/
Tahi Pacific	Processed food products manufacturer	http://www.tahipacific.com
New World IGA	Supermarket	https://www.facebook.com/pages/NEWWORLD-IGA/252868404886324
Morris Hedstrom	Supermarket	http://www.mh.com.fj

For the second component of the study, we conducted ten single semi-structured interviews with open-ended questions with key Fijian stakeholders involved, through their professions, on issues related to public health and diets. We selected participants based on their potential ability to provide detailed insight and first-hand experience into the CPA practices of the food industry in Fiji (purposive sampling). We approached additional participants using a snowball sampling method. Participants included a researcher, two current or former policy makers, six public health advocates (including senior executive officers and other senior staff of government agencies and relevant non-for-profit organisations), and a journalist, all involved in the development and implementation of diet- and public health-related policies. Participants included 8 Fijians and 2 foreign nationals. We did not invite representatives of the food companies to participate in the research as we felt that the commercial perspectives that they were likely to offer would not contribute substantially to the research aim. Participants provided their written informed consent to participate in this study. All data is securely stored at Deakin University, Australia. We pilot tested the interview guide prior to data collection (Additional file 3). We administered interviews face-to-face, at the workplace of participants or in a place of their convenience, and lasted approximately one hour each. With the consent of participants, we digitally recorded discussions and we took field notes during and after each interview. MM conducted and transcribed (semi-verbatim transcription) all interviews. We returned transcripts to participants on request, for their comments and corrections. We managed data using NVivo software. We reached data saturation (i.e., no major new themes emerged from the interviews). We gave a unique identifier code to each participant and we only included these codes in notes and transcripts. Audio recording, codes and consent forms were in an identifiable form, and are stored separate to transcripts and notes. For publication, we only used general identifiers. We employed the generic terms “his” or “he” to refer to both female and male participants.

For both components of the study, MM undertook a qualitative thematic analysis. GS reanalysed the data (inter-coder reliability). We based our initial choice of themes on an existing framework for classifying the CPA of the food industry (as detailed in Table 3) [11]. We used this same framework during interviews, when participants were asked to share their experience and opinion on the CPA of the food industry in Fiji. Through this approach, the framework was used to identify and categorise food industry practices from the data, with the framework adapted, as necessary, based on the findings and emergent themes (iterative process). We reported illustrative examples here, using the framework as a guiding thread.

**Table 3:** Summary of practices identified in Fiji with the systematic approach using publicly available information

Strategy	Practices	McDonald's	Pizza King	Coca Cola Amatil	Tappoo	Motibhai	Frezsco	Pinto	FMF	Foods Pacific	C J Patel	Food processors	Tahi Pacific	New World	Morris Hedstrom	Total occurrences
Information and messaging	Lobbying	0	0	0	0	0	0	0	0	0	0	0	0	0	0	0
	Stress the economic importance of the industry	3	0	3	0	1	0	0	1	0	0	1	0	0	0	9
	Promote de-regulation	0	0	0	0	0	0	0	0	0	0	0	0	0	0	0
	Frame the debate on diet- and public health-related issues	0	0	0	0	0	0	0	0	0	0	0	0	0	0	0
	Shape the evidence base on diet and public health-related issues	0	0	0	0	0	0	0	0	0	0	0	0	0	0	0
Financial incentives	Financial incentives	0	0	0	0	0	0	0	0	0	0	0	0	0	0	0
Constituency building	Establish relationships with key opinion leaders and health organisations	0	0	0	0	0	0	0	0	0	0	0	0	0	0	0
	Seek involvement in the community	1	0	5	4	0	0	0	6	0	3	0	0	0	0	19
	Establish relationships with policymakers	0	0	1	0	0	0	0	0	0	1	0	0	0	0	2
	Establish relationships with the media	1	0	2	0	1	0	0	1	0	4	1	0	0	0	10
Legal strategies	Use legal action (or the threat of) against public policies or opponents	0	0	0	0	0	0	0	0	0	0	0	0	0	0	0
	Influence the development of trade and investment agreements	0	0	0	0	0	0	0	0	0	0	0	0	0	0	0
Policy substitution	Policy substitution	1	0	1	0	1	0	0	1	0	0	1	0	0	0	5
Opposition fragmentation and destabilisation	Opposition fragmentation and destabilisation strategy	0	0	0	0	0	0	0	0	0	0	0	0	0	0	0
Total number of CPA practices identified		6	0	12	4	3	0	0	9	0	8	3	0	0	0	45

## Results

### CPA strategies and practices identified in Fiji

The systematic approach to the collection of publicly-available data (systematic approach) resulted in the identification of 52 relevant documents, 45 of which related to the companies selected for this study. Data analysis revealed the use of three of the CPA strategies by the food industry in Fiji (information and messaging; constituency building; policy substitution) (Table 3). All references and data collected through the systematic approach can be found in Additional file 4, with each piece of information allocated a unique code (starting with the letter A). We identified the most number of CPA activities for McDonald’s, Coca Cola and C J Patel. When supplemented by data from interviews, all six CPA strategies were identified as being used by the food industry in Fiji.

### Information and messaging strategy

Aspects of the information strategy were revealed both through the systematic approach and through interviews. Participants reported that this strategy was probably one of the most influential on public health policies and programs.

#### Lobbying

We did not identify lobbying by the food industry when we implemented the systematic approach. However, half of interviews participants mentioned lobbying as one of the practices that they observed during their careers in public health in the Fijian context. They underlined the fact that lobbying usually goes against efforts made by the Ministry of Health to prevent and control NCDs.

“They [food industry representatives] certainly make direct contact with policy makers to push their own agenda. They will contact the Minister or the Permanent Secretary, or senior civil servants to push their particular agenda – yes most definitely, on a very frequent basis. (…) Generally speaking, that has been negative to public health because they have been pushing or promoting the opposite of what the Ministry of Health is pursuing.”

“This [lobbying] is a subtle technique utilised in the Fijian scene. I know for a fact that the previous Minister of Health introduced bills on increased taxation on sugar sweetened beverages to the Cabinet six times – all were refused. Why? There appears to be some influence on Cabinet by the major [food industry] players.”

The economic importance of the food industry was cited as a potential factor that allowed the food industry to have privileged access to policy makers in Fiji.

“They [the food industry] do lobby policy makers, they do approach the Ministers. But the disadvantage for us [public health advocates] is, for them, they have numbers [of staff], and they have the economic power to lobby, we don’t.”

#### Stress the economic importance of the industry

We found no evidence, from the systematic approach, that the food industry stressed the economic importance of the industry in relation to discussions on NCD prevention. However, this practice was experienced by interview participants who saw it as a major barrier to public health efforts to prevent and control NCDs.

“I would probably say that most influential [practice of the food industry] is their framing of this as being an economic issue.”

When involved in discussions on public health issues in Fiji, interview participants identified that the food industry, for example, stressed the number of jobs supported and the money the industry generated for the economy.

“They [food industry representatives] always remind us that “even though you [public health advocates] are coming from the health side, we still need to feed families.” In other words, “we still provide jobs that support families, and most of all, we provide for the economy of the nation”.”

“Because of how they [the food industry] influence the economy, they feel that they have every right to influence policy makers, or regulation, or anything that is set up by the government. At the end of the day, they feel that, because of their input, they need to be consulted [and shape policy] to what suits them.”

Companies also threatened to withdraw from Fiji, to relocate to other countries in the Pacific or not to provide new employment, when the idea of regulating the food supply to make food environments healthier was raised by governments.

“There have been companies that have said that they are 30 to 40 % contributors to the tax payment to the government, and should there be too much [regulation] from the Ministry of Health, then there is a possibility that the company might close, and so many workers would lose their jobs, or they would shift their plants to some other countries.”

“The response from a number of the industry was that they were not going to open new factories as a result of this [proposed policy measure], and that potential new jobs, hundreds and hundreds of new jobs would no longer be created – which they haven’t created anyway.”

“The lawmakers in the Solicitor’s General office and the Attorney General’s office and the Minister of Trade, they weren’t shifting – because these guys go to them and say, “Hey listen, we’ll close the factories down in Fiji, and we’ll reopen it in Tonga, you’ll lose employment”. So public health suffers at the expense of trade and development.”

#### Promote de-regulation

We did not capture the promotion of de-regulation through publicly available information in Fiji, although many interview participants recalled situations in which the food industry employed this practice when a public health policy was proposed by the Fijian government. Different arguments were used, such as the administrative, trade and economic burden associated with the potential implementation of new policies, or the fact that the Fijian market would not be competitive enough if new policies were considered. However, participants were sceptical about the validity of food industry claims and arguments.

“When we have been asking them to [make changes to food labelling], companies have quoted us that it will cost them FJD 2 million [approx. USD1 million], and they have already ordered ten years’ worth of packets and things like that. But when there are promotional items, [changes to food labelling] just get done overnight.”

#### Frame the debate on diet- and public health-related issues

We found evidence, both through publicly available information and interviews, that the food industry framed the debate on diet- and public health-related issues in Fiji in ways that are favourable to the industry. A newspaper article (A13) described a partnership between the government and the food industry to prevent and control NCDs, where the approach taken emphasised the role of physical activity, individuals and parents rather than the unhealthy food environments that are the primary driver of diet-related NCDs [7].

Interview participants revealed that this practice was commonly employed by the food industry in Fiji. In particular, the soft drinks industry seemed to try and shift the blame away from its products and, rather, to focus the attention of consumers, public health advocates and policy makers on the need to increase physical activity levels of the population.

“If you look at Coca Cola’s approach, then, it’s totally about “Look, you can run around and do this physical activity stuff, and then you can drink and eat as much sugary stuff as you want””

“This has been the major proposal for the sugar sweetened beverages producers –emphasis on increased activity whilst not limiting or restraining their selling pitch.”

The food industry also emphasised the responsibility of individuals in maintaining good health and proposes education as a potential solution to prevent and control NCDs.

“During workshops, consultations, people from the food industry actually say those things. (…) They have shifted the burden, or blame, to the individual. I’ve directly heard this from one of the biggest food manufacturers in Fiji, from their big boss.”

#### Shape the evidence base on diet and public health-related issues

We did not observe the shaping of evidence by the food industry with the systematic approach, but interview participants experienced this practice on many occasions in Fiji. The food industry, in particular Nestlé, supplied education materials to some schools and communities in Fiji. This practice was mostly seen as a marketing strategy by interview participants, where the company would promote its products while presenting the information as coming from public health experts. Interview participants generally viewed this practice as harmful to public health efforts.

“There is Nestlé of course, […] they have a “Kana Vinaka” program. What they basically do is, the National Nutrition and Food Centre has come up with a healthy plate for Fiji, and in the healthy plate it tells you the amount of carbohydrates in pictorial form and they have a healthy pyramid. So what they have done is that they have taken that, and they have translated it in their version. So where there is supposed to be carbs, they put noodles as an option as well. […] They are going village to village, distributing flyers and teaching the community how to make a healthier version of noodles. […] My view on that is, in remote villages, from what we know, they have a very traditional diet practice, so if you are going into a village and you are telling them how to eat a healthier version of noodles, it’s basically… you are telling them to eat noodles.”

“Nestlé were providing nutrition education in schools. They were coming to the schools, talking about good nutrition, how to eat healthy diets, and at the same time, giving children samples. And that involved providing cups and giving children samples of Milo - (…) yeah [cups] with the logo.”

Another practice observed on at least one instance in Fiji was the cherry picking of evidence by the soft drink industry.

“What they [the soft drink company] have done, they created a website on their own beverages. […] They pick up on one or two studies, which have flawed methodology, and they bring it, they share it among themselves, they bring it to conferences, they send us [public health advocates] the papers and they say “Oh but this study is saying this and this [in favour of the company’s products]””

Coca Cola tried to establish an internal working group on diet- and public health-related issues in Fiji, and invited policy makers to join the group.

“You see Coca Cola was trying to do this, they were forming a health committee to look at health, and they said “it’s going to be” [very helpful for public health advocates]… it’s just a hook; it’s a joke you know.”

Moreover, participants mentioned that one of the mechanisms used by the food industry to try and delay the work of public health advocates was to question evidence provided by experts.

### Financial incentives strategy

We did not capture the financial incentives strategy through the systematic approach. This could be explained by the fact that there is currently no disclosure or monitoring of donations to policy makers and political parties in Fiji. Nevertheless, interview participants explained that it could have an important influence on public health policies and programs. Only a few interview participants experienced this strategy during their career in Fiji, although some participants also mentioned that they were not willing to share information about this strategy.

“We know that because they [food companies] fund political parties […]. If you look at some of these major players in the food industry, there is a lot of talk about how they have been influencing and funding political parties over the years, for them to get all of the promotions and so on and so forth. (…) Because of the confidential nature of these things (…), I can’t name them…”

### Constituency building strategy

The constituency building strategy was revealed both through the systematic approach and interviews in Fiji.

#### Establish relationships with key health organisations and opinion leaders

For this study, we did not identify the establishment of relationships between the food industry and key opinion leaders or health organisations for matters related to NCDs.

#### Seek involvement in the community

During the period of data collection, we found evidence of the involvement of the food industry in the community on many occasions with the systematic approach. 19 of the 52 pieces of information we collected were related to this practice.

The Coca Cola Games, an annual Fiji Secondary Schools Athletics Competition, in April 2015, were promoted in the media, before, during and after the Games (Appendix 1: Box 1). FMF also had a Facebook page for its Chow Games, Chow being a brand of noodles sold in Fiji by the company (A24). This is an annual event, held in December for primary schools, and organised by the Fiji Primary Schools Athletic Association (A24). In addition, through its Facebook account, FMF promoted its participation in an oratory program in a primary school for International Women's Day (A21) and in a chess competition (A29). During data collection, FMF supported communities in Vanuatu after the destruction caused by Cyclone Pam, with, for example, a Facebook status update depicting Chow products (A22) and an article in the Fiji Times, one of the local newspapers (A23). We also identified that McDonald’s provided free lunches and face painting to children hospitalised and treated for cancer (A39), and that Tappoo funded education programs on multiple occasions (A48).

Interview participants identified many other examples of programs developed by the food industry in Fiji. As with education materials, interview participants felt that the industry involvement in schools and communities was mainly used to market its products, and they were typically scathing of the food industry’s involvement.

“There is the “Twisties Smokehouse”, which is support given to the fire brigade […]. They have this big inflatable house, which is lovely, and they are taking it into the school, and the kids have to wriggle through and to show how they would escape from a home in the case of smoke and fire. But then, after the lesson, they will get a packet of Twisties. Really?”

“The Milo Mile [outdoor exercise station], I feel at least there is a place for people to exercise and I’ve seen people use it. And they don’t have billboards over there, big billboards over there saying “oh this is Nestlé”, and “eat our products”, “eat more of our products”, or whatever. People probably don’t really even know the name of that thing. They just know it’s a station which they go and use. […] They are putting it in places where people are already walking or exercising. […] You don’t give resources to people who already have access to resources, that’s my argument.”

In addition, the suggestion was made that the food industry gets involved in helping communities in Fiji as a mechanism to divert attention away from their standard business practices.

“They [food companies] say that’s corporate social responsibility, they market it that way. But if you look at what they do on a daily basis, in terms of their trade practices, it’s a different story. We always argue that corporate social responsibility should not be giving donations, but it should be your business practices rather than giving, donating.”

One participant mentioned the fact that the involvement of the food industry in the community is something that is sometimes supported at the international level, as with the sponsorship of the Olympic Games by major food companies, and because of that, it may become challenging for public health advocates to question those relationships at the national level.

#### Establish relationships with policymakers

Both the systematic approach and interviews revealed that the food industry established strong relationships with policy makers in Fiji. The Fijian Government was, for example, collaborating with some major food industry actors to prevent and control NCDs, as reported in the newspaper Fiji Sun.

The Fijian Government has established a partnership with the Fiji Food Industry Group to help combat and reduce non communicable diseases in Fiji. […] These Companies are Nestle Trading Fiji Ltd, Coca-Cola Amatil, Motibhai Group, Food Processors Limited, Flour Mills of Fiji and McDonald’s. […] A joint working group has over seen the implementation of the action plans successfully with the food industry through consultations. This group includes multi-sectorial representation including retailers and the private sectors representatives. […] There has been widespread adoption and acceptance of the initiatives undertaken by the industry. The approach has also fostered a greater working relationship and closer ties with the Government to ensure effective collaboration is based on a partnership rather than the imposition of regulations. (A12, 26, 31, 37, 41, 45)

There were, additionally, many media and government releases promoting the new Free Milk Program launched by the Fijian government in April 2015 (Appendix 2: Box 2).

Moreover, interview participants explained that some companies, in particular large transnationals, had their own staff consulting and influencing government officials.

“They [food industry representatives] bring in their attorneys from the US [United States], from Australia, their nutritionists for New Zealand […] to say “well, you are not doing the right thing”.”

“They [food industry representatives] are closely connected with senior government officials - people like the Prime Minister - and… they become colleagues and then they do some co-work in some other areas. What happens then is the manager or politician takes a soft stance on that [policies for NCD prevention].”

Some interview participants explained that the Ministry of Health, in particular, considered these relationships with the food industry as a strength rather than a threat to the development of public health policies and programs. The Ministry was, indeed, inviting the food industry to be involved in the development of such policies.

“We [the Ministry of Health] work with them [the food industry]. Five years back, there was not any “working with”. The government was that side and the food industry this side, but now, because of the consultations that have happened, we can easily sit together in a room and dialogue and talk. That has been something… a positive, constructive, that has come out from working with these food industries.”

“Our [the Ministry of Health] focus has also shifted towards engaging closer with the industry (…), rather than taking this adversarial kind of relationship. We also try and engage them to come in, and for them to give us their views, for us to share with them our experiences.”

The only interview participant who witnessed the use of the revolving door, which is a situation when an employee moves jobs from one in the food industry to a position in the government (or vice versa), explained that, from his point of view, it could be a strength in terms of capacity building for the government.

Interview participants saw the strong relationships between the food industry and the Ministry of Trade as a major hurdle preventing the development and implementation of public health policies in Fiji.

“The Ministry of Industry and Trade will always progress the initiatives of the food industry. They are there to assist them. So they might not be seeing what we [public health] are seeing. Because health is not their concern, their concern is trade, facilitating trade, at the cost of the health of the people, at the expense of the lives of our children and their health, they don’t see that.”

#### Establish relationships with the media

We identified the majority of the CPA through the systematic approach via the media. Interestingly, most of the relevant newspaper articles were from the Fiji Sun, a newspaper owned by C J Patel. This may indicate some influence of food companies over the information released to the public in Fiji [17]. In addition, interview participants mentioned that there are relationships between food companies and the media. For example, participants explained that journalists get invited to events sponsored by the food industry. These relationships may have been built due to the lack of funds in the media industry, a gap that is filled, in part, by the money paid by food companies to advertise in newspapers and on television.

“[The food industry] do work with [the media] and they are a major source of advertising revenue for media organisations. […] Journalists get invited to launches and launching of products and cocktails and things. […] The link is there with the food industry, the advertising dollar becomes the major factor.”

### Legal strategies

We did not identify legal strategies when using the systematic approach. However, interviews revealed that they were experienced by some participants.

#### Use legal action (or the threat of) against public policies or opponents

One interview participant recalled an event which could serve as an illustration of threats by the food industry to use legal action to prevent harmful publicity.

“We were threatened [a few years ago]. Right at the eleventh hour, the day before we were supposed to go and present [our work], we started receiving faxes from some of the biggest and richest law firms in Fiji, who represent some of the big companies, mostly the local ones – not so much the international, but the local ones, because [what we had to say was compromising for them]. (…) And we had to withdraw our press releases at the eleventh hour. (…), because they actually threatened “if this goes public, we will [sue you].” [We] had to withdraw everything.”

#### Influence the development of trade and investment agreements

Although we did not capture this practice through the systematic approach, and none of the interview participants shared specific examples to illustrate this practice, the influence of the food industry over the development of trade and investment agreements was seen as a positive by one participant.

“They [food company representatives] do lobby and try to influence [trade agreements], but that’s more from protecting our local industry, so it’s not something bad, it’s something good, that we support. They do work, they do raise their concerns in the interests of the local industry, to protect our local industry.”

### Policy substitution strategy

We identified the policy substitution strategy both through the systematic approach and through interviews. It was reported, in the media, that some food companies voluntarily decided to reformulate some of their products and to promote healthy diets, an initiative supported by the Fijian government (A11, 25, 30, 36, 40, 44). However, there was no mention of the specific criteria established for those initiatives, or of their potential evaluation or monitoring.

“”Some of the major food companies in Fiji have undertaken initiatives in voluntarily reducing the levels of sodium, fat and sugar.” […] Specific guidelines are being introduced around marketing of food and beverages for children; this is so that providing children with a healthy diet is fully supported by parents.” (A11, 25, 30, 36, 40, 44)

Many interview participants mentioned examples where the policy substitution strategy was employed to prevent the Ministry of Health from developing or implementing new regulations.

“Well, regulation is always the last thing: that is, if they [the food industry] don’t want to meet us [the government] on equal grounds, then we regulate. But it will be good if they do that themselves, they self-regulate, or voluntarily, but the thing is they are benchmarking according to their own standards. It won’t work.”

A key example of self-regulation was a Memorandum of Understanding (MoU) signed by major soft drink companies in 2013. Participants underlined the lack of evaluation and monitoring of this MoU, and cast doubt over whether it was actually implemented by food companies. Participants suggested that this MoU was signed to prevent the government from introducing a new regulation on marketing of unhealthy food products to children.

“[The Memorandum of Understanding from soft drink companies] was done primarily to try and avert from the path the Ministry of Health has been pursuing, which is to regulate marketing of food to children. […]. And it has been 18/19 months, and there has still been nothing [no action to reduce marketing to children], because there is nothing to report. But it’s a clear stance that they have taken, and they’ve marketed it a lot, and they say that this is their way forward. […] They are supposed to be monitoring that themselves and to be reporting to the Ministry of Health. And the Ministry of Health has written to the group on a number of occasions, saying “Hello! Where is the report?” – nothing.”

Interview participants felt that reformulation of products was primarily a marketing strategy which would have little effect on the prevention and control of NCDs.

“They [the food companies] say they are doing stuff [reformulation of products], but there are ways in which they are doing it. They [do reformulation] on the least consumed products, and they heavily market it, so that becomes marketing in terms of increasing consumption of the product as well.”

The proposed front-of-pack labelling program, which was under development in Fiji, was an example where the Ministry of Health itself proposed a voluntary scheme to food companies. Interview participants explained that the reason for a voluntary, rather than a mandatory scheme was that the Ministry of Health was aware of trade concerns that could be related to the mandatory implementation of this scheme.

“We [the government] just want to go voluntary, because we know that it will have some trade issues and we really want [to not interfere with trade], because Fiji is a growing economy, so we are very mindful of that.”

### Opposition fragmentation and destabilisation strategy

We did not identify the opposition fragmentation and destabilisation strategy through the systematic approach. Interview participants did not raise any specific concern about this strategy, and did not provide evidence of this strategy being used in Fiji.

Only one participant was personally affected by criticism from the food industry and explained that it had a strong impact on his career.

“Why I am sitting here [in this kind of job] today? There were some groups of people [associated with the food industry] who wanted me out [of government]. There were various representations high up the line where they didn’t like the things which were happening”

### Value of monitoring the CPA of the food industry

Most interview participants expressed their interest in identifying and monitoring the CPA of the food industry in Fiji. They were particularly interested to learn more about the practices employed by the food industry in their country, and explained that they might be exposed to the influence of the food industry. Some were concerned that they might not be taking the right approach when dealing with the food industry on diet- and public health-related issues.

“[Information on the practices used by food companies] would be an advantage, not only to the Ministry of Health, but right up to other Ministries. I guess also right up to the community level, that information would be very useful.”

However, most interview participants identified that there is currently limited access to publicly available information in Fiji.

“I guess that the issue would be that certainly here and smaller countries, that we don’t have the rules around the release of public information and things like that. So I’m not sure that you are going to get the information that you would need in order to be able to monitor [the CPA of the food industry]”

“I think as a first level strategy in Fiji, the best information is actually going to come from going and talking to people in those agencies and groups, as well as looking at websites, but there is not going to be as much information as you might like, or as many websites as you might like to support that kind of review.”

## Discussion

This study examined the CPA of the food industry in Fiji, using publicly available information and interviews with key informants.

We identified that the food industry makes use of a diverse number of sophisticated practices that could shape public health-related policy and programs in its favour in Fiji. From both components of the study, it seems that the principal practices used by the major food industry actors in Fiji were the information strategy; establishment of relationships with the community, the media and with policy makers; and the policy substitution strategy. Interview participants were largely scathing of the practices adopted by industry to influence policy, although some participants highlighted perceived benefits of certain industry practices, such as the establishment of relationships between the food industry and the Ministry of Health.

This study provides evidence that, even in remote islands in the Pacific, the food industry takes steps to create a favourable regulatory environment to sell and market their products. Large transnational companies, in particular, appear to have enough resources to target a small market, such as Fiji, with the same CPA practices employed in larger countries. They, for example, sponsored national sporting events, and brought in experts from overseas to consult with governments regarding public health-related policies. However, it is interesting to note that, except from a MoU signed by major sweetened beverages manufacturers/suppliers, the large food companies in Fiji did not combined their resources to operate as a single actor, as they often do in larger countries, such as Australia, where the Australian Food and Grocery Council (AFGC) has a high profile as the representative of many food companies.

Strategies employed by the food industry in Fiji are, in parallel, similar to CPA strategies employed by the tobacco and other industries in other countries [18–22].

This study, to the authors’ knowledge, is the first of its kind to implement a systematic approach to identify and monitor the CPA of the food industry in a middle-income country.

This study makes an important contribution to INFORMAS, as part of efforts to monitor key aspects of food environments related to obesity and NCDs globally. The data collected for this study will supplement similar data collected for INFORMAS in other countries, and, over time, this will enable country and company comparisons and analysis of trends.

However, the study has a number of limitations.

Firstly, with respect to the analysis of publicly-available information, we only collected data related to the monitoring period, and it is not clear how practices vary over time. It is likely that the food industry focuses on different strategies depending on the political climate of the day, pressure from public health groups and other international factors, and this component of the study was not designed to capture these differences. In order to gain a detailed understanding of practices employed by the food industry in a particular country over time, it is likely that a longer period of monitoring, or repeated periods of monitoring, would be needed. Importantly, the key informant interviews provided insight into the practices observed by participants over their whole careers, and thus give a broader understanding of the range of practices adopted by the food industry. Nevertheless, it is possible that some practices identified by participants are no longer employed by the food industry.

Secondly, the list of practices presented in this study is unlikely to be comprehensive. There is currently limited availability of public information related to the CPA of the food industry in Fiji, due to limited disclosure and no existing enabling legislation for many sources of information. Moreover, by their nature, many CPA activities (such as lobbying and relationships between individuals) are not documented. The extent to which the systematic approach to collection and analysis of publicly-available information could be useful in other countries, especially countries with limited disclosure of information, needs to be further investigated. Importantly, key informant interviews appear to be a useful way to supplement the data collected from the systematic approach.

However, the insights provided by interview participants are, by their nature, subjective, difficult to verify, and are also unlikely to represent a comprehensive picture of food industry activities. In particular, in the Fijian context, participants did not feel comfortable discussing all aspects of industry influence (for example, financial incentives offered by the food industry), despite assurance of their confidentiality.

Thirdly, this research did not assess the influence of food industry CPA on policy makers and on decision making processes. Future research should investigate the extent to which these practices impacted on the development and implementation of public health-related policies and programs in Fiji.

## Conclusions

The food industry has used a diverse range of strategies and practices in Fiji that have the potential to influence public health-related policies and programs. From a public health perspective, this is of concern, as they may negatively influence efforts to prevent and control obesity and NCDs in Fiji. There is, therefore, a need for greater transparency from the food industry and from the government in Fiji regarding their policy processes and inter-relationships. For example, routine disclosure of financial contributions made by the industry to Fijian political parties and policy makers would improve transparency. Similarly, a detailed register of lobbyists and formal Freedom of Information legislation that allows public access to government information (including industry submissions to consultations) would strengthen accountability. There is a clear need for public health advocates to campaign for greater disclosure of information from both the government and the food industry in Fiji. This would enable a greater proportion of the CPA of the food industry to be identified. It may also help to ensure that public (rather than industry) interests are favoured as part of the policy process. Indeed, the potential conflict between business interests and public health interests means that further investigation of food industry CPA in other countries is warranted.

## Ethics, consent and permissions

Participants provided their written informed consent to participate in this study.

## Consent to publish

We obtained consent to publish from the participant.

## Appendix

Box 1 The Coca Cola Games

The Coca Cola Games, an annual Fiji Secondary Schools athletics competition, took place during the period of data collection, on Friday 24 and Saturday 25 April 2015. CPA practices related to the Coca Cola Games were identified in four newspaper articles published in the Fiji Times, the Fiji Sun and the Stallion. Interview participants also mentioned examples of CPA practices related to the Coca Cola Games. While the sponsorship of sporting events by food companies is usually studied as a marketing strategy, the analysis provided in Table 4 indicates that Coca Cola may also have used these events to influence communities and policy makers, as part of wider CPA strategies.

**Table 4:** CPA strategies and practices identified related to the Coca Cola Games 2015 in Fiji

CPA strategies and practices	Source	Evidence
Information and messaging: Frame the debate on diet- and public health-related issues	Fiji Times	“Visiting Coca-Cola Amatil managing director Alison Watkins says […] "This is a wonderful opportunity to support and encourage children to develop active and healthy lifestyles. This is also a great opportunity to share the message of keeping Fiji beautiful."”[23]
	Fiji Times	“Coca-Cola Amatil Fiji marketing manager […] said the role of Coca-Cola's sponsorship was to promote an active healthy lifestyle. […] "The games is about enjoyment and about promoting active healthy lifestyles and that's the role of our sponsorship."[24]
	Interview participant	“One is the beverage industry, which supports the biggest athletics meeting in Fiji, which is known as the Coca Cola Games. Their view is they are promoting physical activity, so they are saying that they have indicated the amount of nutrients on the product, in the nutrient panel, and everybody can enjoy a drink, provided you go and run after that. They are trying to promote physical activity through that. […] The trophy itself [at the Coca Cola Games] was like a bottle of Coke, which is advertising for the company and taking it to another level.”
	Interview participant	“Well for Coca Cola, that’s where they are saying that there are sponsored events, they are helping children become active […]. But […] there are structured physical activity classes in the school curriculum, so this is just advertising on their part. Children have always been active. (…)
Financial incentives	Interview participant	“For the Coca Cola Games, I remember, [Coca Cola] gives us a special invitation to come to be part of the Coca Cola Games, and they will be like “oh come, join and see the healthy stuffs that we do with the Coca Cola Games and the PM [Prime Minister] will be there”. […] year after year, they have sent out invitations
Constituency building strategy: Seek involvement in the community	Fiji Sun	“Alison Watkins, Group Managing Director of Coca-Cola Amatil, […] “We are very proud promoting students taking part in athletics,” she said.”[25]
	Fiji Times	“The total sponsorship for the running of the annual Coca-Cola Games from the zones to the finals is in excess of $200,000. This was revealed by Coca-Cola Amatil Fiji marketing manager. […] Financial constraint is the major factor that has affected many of the schools, especially from the maritime zone, which have been forced to fundraise for the trip to the big event because of lack of sponsorship.”[24]
	Interview participant	“A good example is the Coca Cola Games. … It’s targeting school children. They call it the Coke Games, whether they are promoting Coke Zero or what, it’s still Coke. (…) We have been lobbying hard against it…. but […] the Fiji Secondary School Athletics Association, they need the funds to run the competition and Fiji needs to produce those athletes to win gold medals at regional Games, and at Olympics, or wherever they go. (…) Probably there is an alternative, but they are not explored.”
Constituency building strategy: Establish relationships with the media	Fiji Sun	“Alison Watkins, Group Managing Director of Coca-Cola Amatil, “It is a very big event and we thank all the media for the participation and their support over the last couple of months,””[25]
Constituency building strategy: Establish relationships with policymakers	The Stallion	“Sports Minister supports Coca Cola sponsorship for secondary school athletics " “With regards to the issue of Non-Communicable Diseases, it is uncalled for to state that Coca Cola is a major contributing factor to NCDs in the country. There are so many contributing factors to NCDs and it is entirely baseless to call out Coca Cola and tie it in with NCDs; as there are a lot of contributing factors to NCDs and that should be the issue that needs addressing, not the sponsorship. “During the Coca Cola Games itself, the company provides $152,000 towards the successful running of the games on an annual basis and for the last 10 years, this sponsorship has exceeded $1.3 m and this clearly indicates that the company has invested heavily in the sport of secondary school athletics,” he added. […] “To a larger extent, through the Coca Cola sponsorship of the games, it advocates a healthier lifestyle for all interested parties,” said Mr Tuitubou. […] “Coca Cola has been at the forefront of major schools sports sponsorships like the Coca Cola Games and the Coke Zero Deans rugby competition but to put it in another perspective, the thought of Coca Cola contributing to Non-Communicable Diseases is irresponsible.” “We need to find out the root causes of NCDs in the country and not necessarily single out Coca Cola as a contributing factor. The root causes of NCDs is what you put on your plate, and with regards to the issue of obesity, the onus is on the individual to live an active lifestyle in order to prevent that from happening, and this is the very thing that the Coca Cola Games is trying to encourage, a healthier lifestyle.””[26]
	Interview participant	“Let me just go back to Coca Cola – I know the guy who organises these… he is the main co-ordinator of the Coca Cola Games, he is a teacher, and he is employed by the Ministry of Education, but paid for by Coca Cola”
	Interview participant	“The Minister for Sport in Fiji is the person who hands out trophies at the Coke Games. So I’m sure they get on well, and make sure that they are on the good side of the Ministers.”

Box 2 Free Milk Program for Year One students

A Free Milk Program for Year One students was launched by the Fijian government during the period of data collection, at the end of March 2015. It was part of the commitments made by the Fijian Prime Minister during the 2014 election campaign, and involves the provision of a branded bowl and spoon as well as daily free milk and Weet-Bix cereal to all Year One students (aged 6–7 years). The systematic approach was able to identify CPA practices related to the Free Milk Program in two government media releases and three newspaper articles published in the Fiji Sun and in the Fiji Times. It is noted that Fiji Sun is owned by the company C J Patel, a food importer in Fiji, responsible for the distribution of a very broad range of food products, such as Weet-Bix products in Fiji [17]. C J Patel also owns Rewa, a dairy company [17]. Interview participants also identified examples of CPA practices related to the Free Milk Program.

While there are likely to be important health benefits to the Free Milk Program, the analysis provided in Table 5 demonstrates that the Free Milk Program was potentially used by this major food company to strengthen its relationships with the community, the media and policy makers in Fiji. There is a risk that the company will subsequently be in a position to influence other public health policies (potentially involving unhealthy products) in its favour as a result of this initiative.

**Table 5:** CPA strategies and practices identified related to the Free Milk Program for Year One students in Fiji

CPA strategies and practices	Source	Evidence
Constituency building strategy: Seek involvement in the community	Fiji Sun	“Year One students will also be given a serve of Weet-Bix, the Prime Minister announced yesterday while launching the Free Milk Program at the Nakelo District School. […] The program, valued at $420,000, will be providing free Weet-Bix to Year One students along with the free 250 ml of milk per day to schools nationwide. The program is also supported by Sanitarium Australia. CJ Patel Group and Fiji Dairy Marketing Director Nathan Hildebrand said seeing Government’s commitment to Fiji’s future inspired them to do more.”[27]
Constituency building strategy: Establish relationships with the media	Interview participant	“We never said we wanted free milk. (…) It’s an initiative coming right from the top [the Prime Minister]. And they link it up with CJ Patel, which is very closely linked with the media, with the food importers.”
Constituency building strategy: Establish relationships with policymakers	Fiji Sun and media release from the Fijian government	“[The Prime Minister] thanked the CJ Patel Group of Companies and Fiji Dairy Limited who generously provided the Year One students with their own Weet-Bix bowl and spoon. “I wish to thank the CJ Patel Group of Companies and Fiji Dairy Limited who have partnered with my Government for all the hard work it has put in to deliver this massive logistic undertaking and have gone the extra mile to ensure that all Class One students receive a healthy meal every morning,” the Prime Minister said.”[27]
	Interview participant	“Take the example of the “Free Milk Initiative” recently, how come we got to put Weetabix onto it? Fiji will never be able to locally grow wheat; therefore Weet-Bix will always be imported. When you are going to put Weet-Bix and milk today, then it will have to continue for years to come. For one year, CJ Patel might say “Ok, I’ll provide you the Weetabix”, when you expand the program, then they will say “I’m running low on money, I need money now”. We will perpetually need their Weet-Bix. Why couldn’t we have put two bananas there, or local foods? (…) For example, bananas are grown all over the country side, so they can buy it locally. The money which they pay for the cereals could have gone to the farmer, so the farmer has a few dollars in his pocket, and he is going to the shop, … so there will be exchanges going on locally, rather than all going out for an exchange… So those sorts of things are happening, yes. (…) It came as part of the election, at the top.”

## Additional files

Additional file 1: Description of CPA strategies (DOCX 21 kb) Additional file 2: Sources of information and searches conducted in Fiji (DOCX 35 kb) Additional file 3: Interview guide (DOCX 28 kb) Additional file 4: Data retrieved during data collection in Fiji (DOCX 63 kb)
