# Supplementary material for: Analysis of the corporate political activity of major food industry actors in Fiji
Source: Global Health. 2016 May 10;12:18. doi: 10.1186/s12992-016-0158-8 (PMC4862126; doi:10.1186/s12992-016-0158-8)
Supplement: Additional file 1: — Description of CPA strategies (DOCX 21 kb) [file 12992_2016_158_MOESM1_ESM.docx]

# Supporting Information 1 - Description of CPA strategies, from Mialon et al. [1]

The information strategy includes practices through which the industry disseminates information that is beneficial to its activities in order to influence public health policies and outcomes in a way that would favour corporations. This strategy includes: lobbying; stressing the economic importance of the industry; promoting de-regulation; framing the debate on diet- and public health-related issues; shaping the evidence base on diet and public health-related issues.

Through the financial incentives strategy, the industry provides funds, gifts and other incentives to politicians, political parties and other decision makers.

The aim of the constituency building strategy is to gain the favour of public opinion as well as other stakeholders such as the media and the public health community. This strategy includes: establishing relationships with key opinion leaders and health organisations; seeking involvement in the community; establishing relationships with policymakers; establishing relationships with the media.

When threatened by regulation, the industry used legal action (or the threat of) against public policies or opponents. It also influenced the development of trade and investment agreements.

Finally, the constituency fragmentation and destabilization strategy refers to the practices employed by the industry to prevent and counteract criticism of a company’s products or practices.

1. Mialon M, Swinburn B, Sacks G: **A proposed approach to systematically identify and monitor the corporate political activity of the food industry with respect to public health using publicly available information**. *Obesity Reviews* 2015, **16**(7):519-530.
