# Supplementary material for: Analysis of the corporate political activity of major food industry actors in Fiji
Source: Global Health. 2016 May 10;12:18. doi: 10.1186/s12992-016-0158-8 (PMC4862126; doi:10.1186/s12992-016-0158-8)
Supplement: Additional file 2: — Sources of information and searches conducted in Fiji (DOCX 35 kb) [file 12992_2016_158_MOESM2_ESM.docx]

Supporting Information 2: Sources of information and searches conducted in Fiji, , based on methods developed by Mialon et al. [1]

| **Nature of the source of information** | **Category** | **Source of information** | **Specific data to be collected** |
| --- | --- | --- | --- |
| Food industry material | Country-specific website of the industry actor | **Mc Donald's:** http://www.mcdonaldsfiji.com/ **Pizza King:** http://www.pizzaking.com.fj/about-us.html  **Coca Cola:** http://www.ccamatilfiji.com/ **Tappoo:** http://www.tappoo.com.fj/ **Motibhai:** http://www.motibhai.com/  **Frezco:** http://www.frezcofiji.com/ **Pinto:** http://pintoindustries.com.fj/index.html  **FMF:** http://www.fmf.com.fj/ **Foods Pacific:** http://foodspacific.com/ **C J Patel:**  Southern Cross Foods (subsidiary Fiji) http://www.scfoods.com.fj/  **Food Processors:** http://www.foodprocessors.com.fj/ **Tahi Pacific:** http://www.tahipacific.com  **MH:** http://www.mh.com.fj | • Composition of diet-related committee • Webpages, reports related to diet-related issues • Voluntary initiatives, commitments and policies related to diet-related issues • Awards to researchers • Research units or groups on diet-related issues • Submissions to public consultations  • Education material about diet-related issues  • Qualitative analysis for information relevant to the conceptual framework [1] |
|  | Country-specific social media accounts of the industry actor | **PAFCO:** https://www.facebook.com/pages/Pacific-Fishing-Company-Limited/555403491175417 - no new messages since 2013  **FMF Foods**:  https://www.facebook.com/FMFFoods  **Foods Pacific:** https://www.facebook.com/foodspacificltd  **New World IGA Supermarket:** https://www.facebook.com/pages/NEWWORLD-IGA/252868404886324  **Pizza King:** https://www.facebook.com/pages/Pizza-King-Wishbone/192340250863561 - no new messages since Aug 2014 | Social media accounts were monitored daily from March to May 2015: Qualitative analysis for information relevant to the conceptual framework [1] |
|  | Country-specific webpages or report or information in annual reports of a company's philanthropic activities | **Motibai:** http://www.motibhai.com/About-Motibhai/Corporate-Responsibility.aspx | Qualitative analysis for information relevant to the conceptual framework [1] |
| Government material: departments (and related agencies) responsible for diet- related issues | Websites of departments and related agencies in charge of health (National level) | Governement: http://www.fiji.gov.fj Ministry of Health: http://www.health.gov.fj/ National Food and Nutrition Center: http://www.nutrition.gov.fj/ | - 2012 - 2014 submissions to public consultations from the food industry and its allies (including third parties) on diet- and public health-related issues - Consultations include: nutrition, health and related claims, dietary guidelines and obesity - Qualitative analysis for information relevant to the conceptual framework [1] - Working groups on diet-related issues and conflicts of interest Public private initiatives |
|  | Websites of the Parliament and Senate (National level) | www.parliamentlive.gov.fj/ Senate abolished in 2013 | 2012 - 2014 Submissions to public consultations from the food industry and its allies (including third parties such as front groups) on diet- and public health-related issues - Qualitative analysis for information relevant to the conceptual framework [1] |
|  | Pacific Islands Forum Secretariat Secretariat of the Pacific Community | http://www.forumsec.org.fj/ http://www.spc.int/ | Qualitative analysis for information relevant to the conceptual framework [1] |
|  | Websites of 3 major political parties (National level) | www.electionsfiji.gov.fj  **Social Democratic Liberal Party:** https://www.facebook.com/sodelpa **FijiFirst:** fijifirst.com  https://www.facebook.com/FijiFirstOfficial **National Federation Party:** nfpfiji.com  https://www.facebook.com/nfpfiji | 2012 - 2014 Annual returns for donations from the food industry (donations for elections and donations to political parties, does not include Public Relations agencies, which are not exclusively working on behalf of the food industry) |
| Other material | Websites of up to 10 major universities with a School/Department of nutrition/dietetics/exercise or physical activity | Fiji National University - http://www.fnu.ac.fj | Searches on the websites and asked (to vice chancellor, deputy vice chancellor (research) and research services) for   - Funds received or sponsor from the food industry - Research projects, fellowships or grants funded by the selected food industry actors - Prizes to students |
|  |  |  | Qualitative analysis for information relevant to the conceptual framework [1] |
| Media | News and media releases | Google News http://www.fiji.gov.fj: media centre | Monthly monitoring from March to May 2015: Qualitative analysis for information relevant to the conceptual framework [1] |
|  |  | **Newspapers:** http://www.fijitimes.com/ http://fijisun.com.fj/ http://fijivillage.com/ | Daily monitoring from March to May 2015: Qualitative analysis for information relevant to the conceptual framework [1] |

1. Mialon M, Swinburn B, Sacks G. A proposed approach to systematically identify and monitor the corporate political activity of the food industry with respect to public health using publicly available information. Obesity Reviews. 2015;16(7):519-30. doi:10.1111/obr.12289.
