# Supplementary material for: Analysis of the corporate political activity of major food industry actors in Fiji
Source: Global Health. 2016 May 10;12:18. doi: 10.1186/s12992-016-0158-8 (PMC4862126; doi:10.1186/s12992-016-0158-8)
Supplement: Additional file 3: — Interview guide (DOCX 28 kb) [file 12992_2016_158_MOESM3_ESM.docx]

# Supporting Information 3: Interview guide

***Thank you*** *for agreeing to take part in this research project that forms a critical part of my PhD in public health. I really appreciate you allocating the time to contribute to this important project.*

***Skype:*** *Are you in* ***a private space****, so we can protect your confidentiality?*

*I expect that the interview will take about 45 minutes to one hour.*

*The context of this research is the prevention and control of non-communicable diseases, such as diabetes and cancers, as well as obesity.*

*The power and influence of the food industry has been identified as a major factor that influences public health policies, directly or not, around the world.*

*We are aiming to develop an approach to systematically identify, understand and monitor these practices.*

*I will use the term “food industry” here to refer to:*

*large packaged food manufacturers,*

*food retailers,*

*soft drink companies,*

*fast food restaurants,*

*as well as food growers,*

*trade unions,*

*public relations firms,*

*and other individuals or groups affiliated with food companies.*

*You have been selected because of your first-hand, in-depth experience observing and/or interacting with the food industry, and we believe you can provide critical insight into the practices of the food industry. Do you have any question so far?*

*I will* ***transcribe*** *this interview myself, and, if you are interested, I can send you the transcript. All data related to this interview will be* ***securely stored at Fiji National University and then transferred to Deakin University, for a period of 6 years after I have analysed all interviews and reported on the findings in my PhD thesis and in scientific journals****. Please be 100% assured that all the information you provide in this interview will not be linked to your name and it will not be identifiable as having been provided by you.*

***Do you have any question about the Plain Language Statement form I sent to you, or about this study****?*

***I will record the interview*** *(****Skype: no video recording****)****, and take notes****, to make sure I accurately record your views. Please do not hesitate to ask me if you want the recording to be stopped.*

***Sign the consent form*** *if not done yet.*

*I am checking my recorder.*

*I have got my questions here; can I start by asking you…?*

*Key questions Prompts (if necessary)*

- Could you tell me which kind of interactions you have with the food industry, as part of **your profession**?

*Current job, previous jobs, meetings, partnerships, funds*

- And specifically, in what **professional roles** and in what situations have you interacted/observed the food industry or their representatives?
- Do you feel that any of the events/interactions you have mentioned may pose a risk to public health? Why?
- There is a strong body of evidence, in the literature, that some of the practices of the food industry may influence public health policies, directly or indirectly. I have a list of such practices here. *Present the strategies (separate document) one by one.*
  - Looking at that list, can you indicate whether you have experienced or observed these practices? I am really interested in **your own professional experience** here. Do you feel these practices may pose a risk to public health and why?

*Present practices identified in the literature*

- - Is there **any other practice** that you have observed, and that is not mentioned here?
  - Could you please indicate which of these practices you feel are **most influential** on public health? And which are **least influential**?
- Now, I am moving to another part of our research project, which seeks to systematically identify and monitor all these practices, using publicly available information only – in order to increase the transparency and accountability of the food industry.
  - Do you think that could be **useful**? Why?
  - What recommendations do you have on **how to identify the practices** you have experienced/observed other than by asking individuals in your position?

*Websites, registers, conferences*

- Finally, we would like to interview other people who can provide insight into this area. Who else do you suggest we interview about this?

*e.g., ex-politicians, ex-food industry people, other people who have extensive experience in this area*

- Is there anything else you would like to talk about?

***Thank you again*** *for your valuable insight into this area and for offering your time to help with this research project. I am very grateful for your help.*

*Turn off the recorder.*
