# Supplementary material for: Analysis of the corporate political activity of major food industry actors in Fiji
Source: Global Health. 2016 May 10;12:18. doi: 10.1186/s12992-016-0158-8 (PMC4862126; doi:10.1186/s12992-016-0158-8)
Supplement: Additional file 4: — Data retrieved during data collection in Fiji (DOCX 63 kb) [file 12992_2016_158_MOESM4_ESM.docx]

Supporting Information 4: Documents retrieved during data collection

| **Reference in manuscript** | **Food industry actor** | **Source** | **Strategy** | **Practice (code used for analysis)** | **Data coded** | **Notes** | **Website URL** |
| --- | --- | --- | --- | --- | --- | --- | --- |
| A1 | C J Patel | Industry | Constituency building | Establish relationships with the  media | Fiji Sun owned by CJ Patel, who also own the dairy company and import Sanitarium C J Patel owns Razor (AdWorks), a communications & advertising agency speacialising in marketing, corporate communications and media buying. Apart from the basket of CJP brands, the company's portfolio includes multinational accounts such as  Coca-Cola |  | http://cjp.com.fj/ |
| A2 | C J Patel | News | Constituency building | Establish relationships with policy makers | As part of their election strategy, the Fiji First Party promised free milk for Class One students throughout Fiji. Currently, the Bainimarama Government is delivering on the promise and the milk, no doubt to the great delight of Rewa Dairy Company and its owner, CJ Patel. Whether this is the best way to use tax payers’ funds to improve children’s nutrition and education, I leave to the government nutritionists to answer (if they ever bother). But astonishingly, the Bainimarama Government also announced that with the milk, they were also handing out Weet bix and special branded bowls and spoons to go with it. The Minister of Education (Dr Mahendra Reddy) and the Prime Minister (Bainimarama) duly made the presentations at some village school, accompanied of course by the ubiquitous salusalu, and grateful thanks from the villagers. Handouts, of any kind are always welcome, anywhere in Fiji. But, was the Ministry of Health consulted on this Weet bix initiative? Of course, the supplying company Sanitarium, and their agents in Fiji, would have been delighted to give it “for free”, but no doubt hoping that the consumption of that product will go up in future, as it is likely to do, especially with the Ministers’ blessings. But most Fiji families have good local breakfast foods, which the Wheetbix is substituting. If our children get used to Weet bix, of course, they will not want to eat dalo or kumala or yams or some roti and curry, for breakfast. The Weet bix is a more expensive and imported product, whatever its nutritional value. If the trend continues, Fiji will be importing more of such products, worsening our food security, and probably worsening the children’s nutrition, All with the blessing and assistance of the Minister of Education and the Prime Minister, of course. |  | http://www.fijitimes.com/story.aspx?id=300682 |
| A3 | C J Patel | News | Constituency building | Establish relationships with policy makers | Year One students will also be given a serve of Weet-Bix, **the Prime Minister** announced yesterday while launching the free milk programme at the Nakelo District School. He thanked the CJ Patel Group of Companies and Fiji Dairy Limited who generously provided the Year One students with their own Weet-Bix bowl and spoon. “I wish to thank the CJ Patel Group of Companies and Fiji Dairy Limited who have **partnered with my Government** for all the hard work it has put in to deliver this massive logistic undertaking and have gone the extra mile to ensure that all Class One students receive a healthy meal every morning,” the Prime Minister said. **He paid tribute to the late managing director of CJ Patel, Sundip Patel, for passionately sharing and giving meaning to this vision.** | Rewa logo can be seen in the background | http://fijisun.com.fj/2015/03/24/weet-bix-treat-for-kids/ |
| A4 | C J Patel | Government | Constituency building | Establish relationships with policy makers | HON PM BAINIMARAMA SPEECH AT LAUNCH OF THE FREE MILK PROGRAM AT DAMA DISTRICT SCHOOL IN BUA PROVINCE (...) When I launched the national Free Milk Program for Year One students in Nakelo on Viti Levu two weeks ago, it was one of my happiest days as Prime Minister. (...)You children in Dama are joining more than 20,000 Year One children across Fiji who are benefiting from this program- a 250 millilitre package of free milk plus a serve of Weet-bix to give you a nutritious breakfast every day of the school week.  It is a partnership between my Government and the CJ Patel Group and Fiji Dairy Limited, who have generously provided the Weet-bix and your bowls and spoons. So I want to thank CJ Patel and especially its former Managing Director, Mr. Sandip Patel - who recently passed away - for their generosity and for sharing our vision to give every Year One student the best possible start to their day. (...) To every Year One student I say; enjoy your milk and your Weet-bix. I can tell from your smiles and your beaming faces how much you appreciate what you have been given. But also use it to stay fit and healthy in every other aspect of your daily lives. Don’t eat sweets - or too many sweets. Eat more fruit. Don’t drink soft drink- or too much soft drink. Drink water or fresh fruit juice and drink milk. And eat more fresh fish and fresh vegetables because those are the best foods to make you big and strong. And always remember this; that to keep fit you need to do as much exercise as you can. Get hold of a ball and kick or pass around. Run as much as you can. And make sure you get involved in those sporting activities your school or community offers you. Use this free milk and Weetbix to start your day and get plenty of exercise. And you can avoid some of the health problems many older Fijians are suffering because they haven’t looked after themselves properly. |  | http://www.fiji.gov.fj/Media-Center/Speeches/HON-PM-BAINIMARAMA-SPEECH-AT-LAUNCH-OF-THE-FREE-MI.aspx |
| A5 | C J Patel | Government | Constituency building | Establish relationships with policy makers | HON. PM BAINIMARAMA SPEECH AT THE LAUNCH OF DISTRIBUTION OF FREE MILK (...) I’m delighted to be with you all today to launch a landmark initiative to improve the health of our children - the Free Milk Program for Year One students at schools throughout Fiji. (...) I’m especially delighted to announce that as well as a free glass of milk a day, each Class One child will also be given a serve of Weetbix a day, thanks to the CJ Patel Group and Fiji Dairy Limited. They are also very generously providing you with your very own bowl and spoon. I wish to thank the CJ Patel Group of Companies and Fiji Dairy Limited who have partnered with my Government for all the hard work it has put in to deliver this massive logistic undertaking and have gone the extra mile to ensure that all class one students receive a healthy meal every morning. I also wish to, in particular pay tribute to former Managing Director, Mr. Sandip Patel, who recently passed away, for passionately sharing and giving meaning to this vision. Our big Vinaka vakalevu. As many of you know, Weetbix is a nutritious breakfast cereal that is high in fibre and low in sugar. And it contains many of the vitamins and minerals that young people also need to give them energy and the best possible start to the day. One Weetbix and a 250 millilitre packet of milk every school day is a tremendous boost to the health and well-being of our Year One children.  (...) Children, enjoy your milk and your Weetbix. It will give you a wonderful start to the day and help give you a wonderful start in life. I also want you all to think carefully about staying fit and healthy in every other aspect of your daily lives. Don’t eat sweets - or too many sweets. Eat more fruit. Eat Weetbix. Don’t drink soft drink- or too much soft drink. Drink water or fresh fruit juice and drink milk. And eat more fresh fish and fresh vegetables because those are the best foods to make you big and strong. And always remember this; that to keep fit you need to do as much exercise as you can. Get hold of a ball and kick or pass it around. Run as much as you can. And make sure you get involved in those sporting activities your school or community offers you. It doesn’t matter if you’re not great at sports. The important thing is to play in the right spirit, learn the benefits of team work, and get the exercise you need to grow into a healthy adult. Because if you don’t eat well and exercise, you can get some very serious illnesses. Use this free milk and Weetbix to start your day and get plenty of exercise. And you can avoid some of the health problems that many older Fijians are suffering because they haven’t looked after themselves properly. (...) I now have great pleasure to launch the Government’s Free Milk Initiative now also with free Weetbix. And to Year One students all over Fiji I say: Drink up your milk everybody and of course eat your Weetbix. And here’s to a happy and healthy life for you all. | CJ Patel also owns the dairy company and imports Sanitarium | http://www.fiji.gov.fj/Media-Center/Speeches/HON--PM-BAINIMARAMA--SPEECH-AT-THE-LAUNCH-OF-DISTR.aspx |
| A6 | C J Patel | News | Constituency building | Seek involvement in the community | The Nakelo Rugby Club received a timely donation of jerseys from CJ Patel Group at Nauluvatu Village, Nakelo, Tailevu yesterday. (…) Also at the handover was Tailevu Rugby president Maika Rainima who said that **CJ Patel’s contribution had impacted not just a team but the whole district.** “This has obviously boosted the team very much because most of our young boys have finished school and are looking for jobs,” he said. “CJ Patel Group has not only impacted just a group of boys but a whole district, a whole generation. (...) CJ Patel’s national sales manager Khem Raj said they planned on continuing such donations in the future. **“We are known for sponsoring major tournaments such as the Skipper Cup but we now want to take it another level,” Raj said. “We want to help villages and clubs in future.”** | Fiji Sun owned by CJ Patel | http://fijisun.com.fj/2015/05/15/nakelo-roosters-thankful/ |
| A7 | C J Patel | News | Constituency building | Seek involvement in the community | Year One students will also be given a serve of Weet-Bix, the Prime Minister announced yesterday while launching the free milk programme at the Nakelo District School. He thanked the **CJ Patel Group of Companies and Fiji Dairy Limited who generously provided the Year One students with their own Weet-Bix bowl and spoon.** “I wish to thank the CJ Patel Group of Companies and Fiji Dairy Limited who have partnered with my Government for all the hard work it has put in to deliver this massive logistic undertaking and have gone the extra mile to ensure that all Class One students receive a healthy meal every morning,” the Prime Minister said. He paid tribute to the late managing director of CJ Patel, Sundip Patel, for passionately sharing and giving meaning to this vision. The programme, valued at $420,000, will be providing free Weet-Bix toYear One students along with the free 250 millilitres of milk per day to schools nationwide. The programme is also supported by Sanitarium Australia. CJ Patel Group and Fiji Dairy Marketing Director Nathan Hildebrand said seeing Government’s commitment to Fiji’s future inspired them to do more. | Video and photo of the launch for the event - advertisement signs for the Rewa company - Fiji Sun owned by CJ Patel, who also owns the dairy company and imports Sanitarium https://www.youtube.com/watch?feature=player_embedded&v=rg01Wuanbo0 | http://fijisun.com.fj/2015/03/24/weet-bix-treat-for-kids/ |
| A8 | C J Patel | News | Constituency building | Seek involvement in the community | Nothing could explain the joy and smiles on the faces of the Year One students of Lakeba in Lau when they received their first batch of free Rewa Life milk and Weet-Bix from Government.(…) The Government’s distributation of Rewa Life milk and Weet-Bix is supported by C.J. Patel and Fiji Dairy Limited. | Picture with the Ministry and a bottle of branded milk in front of kids - Fiji Sun owned by CJ Patel, who also own the dairy company and import Sanitarium | http://fijisun.com.fj/2015/05/02/islanders-praise-free-rewa-life/ |
| A9 | Coca | Facebook | Constituency building | Seek involvement in the community | Coca Cola Games: Fiji Secondary Schools Athletics Competition | Logos of Coca on pictures (stadium, athlets, etc) | https://www.facebook.com/cocacolagamesfiji |
| A10 | Coca | News | Constituency building | Seek involvement in the community | Ministry of Education and Sports In most developed countries, the ministries of education and government will not allow the manufacturers of junk food to sponsor school events. But it is perfectly OK in Fiji. For a few years now, the national athletic championships for schools has been called after a soft drink, Coca Cola, largely because the private soft drink company provides some funding which government is not willing to provide. The inevitable result is that the company brand name is thrust into children’s minds day in day out, and through out the year, associated with a healthy sporting exercise. The company brand name is mentioned dozens of times over television news, while the company representative enthuses about the passion that schools, teachers and students, are showing the Games. Instead, what the Ministry of Education does indirectly, is encourage the consumption of an incredibly sweetened drink, whose excessive consumption is associated with diabetes in our children. Often, meal packages from popular food outlets, include that product together with a hamburger or fish, with chips. Is it any wonder that with such official ministerial support, the consumption of that product keeps increasing, as does the incidence of diabetes (...) With the blessing of the Ministry of Education, the Coca Cola Games torch is now carried throughout hundreds of schools, with students and teachers lined up to honor it and implicitly, the product of course. While the soft drink vending machines are everywhere, there is little attempt to ensure that clean piped water is available for the thousands of children to drink as the better healthier thirst quenching drink. |  | http://www.fijileaks.com/home/health-of-nation-under-threat-one-ministry-builds-and-another-destroys-worsening-nutrition-in-fiji-professor-wadan-narsey |
| A11 | Coca | News | Policy substitution | Policy substitution | The Fijian Government has established a partnership with the Fiji Food Industry Group to help combat and reduce Non communicable diseases in Fiji. While speaking at the World Consumer Rights Day yesterday, the Minister for Industry Trade and Tourism, Faiyaz Siddiq Koya addressed the initiatives that were going to be put in place. He said: “Some of the major food companies in Fiji have undertaken initiatives in voluntarily reducing the levels of sodium, fat and sugar.” These Companies are Nestle Trading Fiji Ltd, Coca-Cola Amatil, Motibhai Group, Food Processors Limited, Flour Mills of Fiji and McDonald’s. The Fiji Food Industry Group has holistically committed to a plan of action that ensures a co-ordinated and comprehensive approach towards this initiative. Specific guidelines are being introduced around marketing of food and beverages for children. | C J Patel (Fiji Sun) also owns Razor (AdWorks), a communications & advertising agency speacialising in marketing, corporate communications and media buying. Apart from the basket of CJP brands, the company's portfolio includes multinational accounts such as  ANZ Bank  British American Tobacco  Coca-Cola  Colgate Palmolive | http://fijisun.com.fj/2015/03/25/healthy-living-is-priority-koya/ |
| A12 | Coca | News | Constituency building | Establish relationships with policy makers | The Fijian Government has established a partnership with the Fiji Food Industry Group to help combat and reduce Non communicable diseases in Fiji. These Companies are Nestle Trading Fiji Ltd, Coca-Cola Amatil, Motibhai Group, Food Processors Limited, Flour Mills of Fiji and McDonald’s. A joint working group has over seen the implementation of the action plans successfully with the food industry through consultations. There has been widespread adoption and acceptance of the initiatives undertaken by the industry. “The approach has also fostered a greater working relationship and closer ties with the Government to ensure effective collaboration is based on a partnership rather than the imposition of regulations, “This collaborative approach is a key to continuous and sustainable change in the environment for promotion of a healthier Fiji for a while providing consumers accessibility to healthy food,” explained Mr Koya. | C J Patel (Fiji Sun) also owns Razor (AdWorks), a communications & advertising agency speacialising in marketing, corporate communications and media buying. Apart from the basket of CJP brands, the company's portfolio includes multinational accounts such as  ANZ Bank  British American Tobacco  Coca-Cola  Colgate Palmolive | http://fijisun.com.fj/2015/03/25/healthy-living-is-priority-koya/ |
| A13 | Coca | News | Information and messaging | Frame the debate on diet- and public health-related issues | The Fijian Government has established a partnership with the Fiji Food Industry Group to help combat and reduce Non communicable diseases in Fiji. These Companies are Nestle Trading Fiji Ltd, Coca-Cola Amatil, Motibhai Group, Food Processors Limited, Flour Mills of Fiji and McDonald’s. Companies who are part of the group are dedicated to proactively promote healthy active lifestyles and diets on the marketing side as well. Mr Koya highlight: “A key issue in Fiji is low intake of fruits and vegetables and high intake of starchy foods, most of which are not manufactured "Working on helping consumers change their eating patterns to a more holistic manner is the key to long-term success and integral to the efforts of the food industry." Specific guidelines are being introduced around marketing of food and beverages for children, this is so that providing children with a healthy dies is fully supported by parents. | C J Patel (Fiji Sun) also owns Razor (AdWorks), a communications & advertising agency speacialising in marketing, corporate communications and media buying. Apart from the basket of CJP brands, the company's portfolio includes multinational accounts such as  ANZ Bank  British American Tobacco  Coca-Cola  Colgate Palmolive | http://fijisun.com.fj/2015/03/25/healthy-living-is-priority-koya/ |
| A14 | Coca | News | Constituency building | Establish relationships with the media | The Coca-Cola Games torch made its final journey from Nadi International Airport yesterday. (….) This torch was then relayed to Alison Watkins, Group Managing Director of Coca-Cola Amatil. Visiting Fiji for the first time, Watkins was amazed by the enthusiasm showed by the participants. “Coca-Cola has been involved for 41 years and I’m just incredibly proud to see the level of enthusiasm that goes into the games,” Watkins said. (...) Coca-Cola Amatil sales manager Lawrence Tikaram said the **students of Ratu Navula College and Votualevu College would escort the torch to the Coca-Cola Amatil factory in Suva**. “It is a very big event and we thank all the media for the participation and their support over the last couple of months,” Tikaram said. “It has defiantly been an engaging and enlightening moment for all of us, we all have been inspired by the thousands of athletes who have prepared well for the games. We are sure the whole of Fiji will be watching and most importantly we want you to come out and support your students.” | C J Patel (Fiji Sun) also owns Razor (AdWorks), a communications & advertising agency speacialising in marketing, corporate communications and media buying. Apart from the basket of CJP brands, the company's portfolio includes multinational accounts such as  ANZ Bank  British American Tobacco  Coca-Cola  Colgate Palmolive | http://fijisun.com.fj/2015/04/24/i-am-very-proud-says-watkins/ |
| A15 | Coca | News | Constituency building | Seek involvement in the community | The Coca-Cola Games torch made its final journey from Nadi International Airport yesterday. (….) This torch was then relayed to Alison Watkins, Group Managing Director of Coca-Cola Amatil. Visiting Fiji for the first time, Watkins was amazed by the enthusiasm showed by the participants. (...) “We are very proud promoting students taking part in athletics,” she said. (...) | C J Patel (Fiji Sun) also owns Razor (AdWorks), a communications & advertising agency speacialising in marketing, corporate communications and media buying. Apart from the basket of CJP brands, the company's portfolio includes multinational accounts such as  ANZ Bank  British American Tobacco  Coca-Cola  Colgate Palmolive | http://fijisun.com.fj/2015/04/24/i-am-very-proud-says-watkins/ |
| A16 | Coca | News | Constituency building | Seek involvement in the community | VISITING Coca-Cola Amatil managing director Alison Watkins says her message, to athletes at this years Coca-Cola Games is, "strive for your personal best and if you've enjoyed yourself, you've won." Watkins, who arrived in the country on Tuesday evening, said she was happy to be involved in projects Coca-Cola Amatil supports. "One of the favourite parts of my role is to be involved in community projects Coca-Cola Amatil supports and experience the difference they make to the community we are part of," Watkins said. "Coca-Cola Amatil's involvement in the Coca-Cola Games spans over 40 years and is now one of the largest high school events in Fiji. |  | http://www.fijitimes.com/story.aspx?id=303261 |
| A17 | Coca | News | Information and messaging | Frame the debate on diet- and public health-related issues | VISITING Coca-Cola Amatil managing director Alison Watkins says her message, to athletes at this years Coca-Cola Games is, "strive for your personal best and if you've enjoyed yourself, you've won." (...) "This is a wonderful opportunity to support and encourage children to develop active and healthy lifestyles. This is also a great opportunity to share the message of keeping Fiji beautiful." |  | http://www.fijitimes.com/story.aspx?id=303261 |
| A18 | Coca | News | Information and messaging | Frame the debate on diet- and public health-related issues | THE total sponsorship for the running of the annual Coca-Cola Games from the zones to the finals is in excess of $200,000. This was revealed by Coca-Cola Amatil Fiji marketing manager Lawrence Tikaram at a press conference at the Nadi airport yesterday. Tikaram said the **role of Coca-Cola's sponsorship was to promote an active healthy lifestyle.** (...) "The **games is about enjoyment and about promoting active healthy lifestyles and that's the role of our sponsorship.**" |  | http://www.fijitimes.com/story.aspx?id=303263 |
| A19 | Coca | News | Constituency building | Seek involvement in the community | THE total sponsorship for the running of the annual Coca-Cola Games from the zones to the finals is in excess of $200,000. This was revealed by Coca-Cola Amatil Fiji marketing manager Lawrence Tikaram at a press conference at the Nadi airport yesterday. (...) "We fund the total running from the zones to the finals," he said. "It would be in excess of $200,000 in the support we provide from day one. But the most important thing is that **the gate takings go to the Fiji Secondary Schools Athletics Association.**" (...) Financial constraint is the major factor that has affected many of the schools especially from the maritime zone which have been forced to fundraise for the trip to the big event because of lack of sponsorship. |  | http://www.fijitimes.com/story.aspx?id=303263 |
| A20 | Coca | News | Constituency building | Establish relationships with policy makers | SPORTS MINISTER SUPPORTS COCA COLA SPONSORSHIP FOR SECONDARY SCHOOL ATHLETICS " “**With regards to the issue of Non-Communicable Diseases, it is uncalled for to state that Coca Cola is a major contributing factor to NCDs in the country. There are so many contributing factors to NCDs and it is entirely baseless to call out Coca Cola and tie it in with NCDs; as there are a lot of contributing factors to NCDs and that should be the issue that needs addressing, not the sponsorship.** “During the Coca Cola Games itself, **the company provides $152,000 towards the successful running of the games on an annual basis and for the last 10 years, this sponsorship has exceeded $1.3m and this clearly indicates that the company has invested heavily in the sport of secondary school athletics**,” he added. (...) “**To a larger extent, through the Coca Cola sponsorship of the games, it advocates a healthier lifestyle for all interested parties**,” said Mr Tuitubou. (...) “Coca Cola has been at the forefront of major schools sports sponsorships like the Coca Cola Games and the Coke Zero Deans rugby competition but to put it in another perspective, **the thought of Coca Cola contributing to Non-Communicable Diseases is irresponsible**.” “**We need to find out the root causes of NCDs in the country and not necessarily single out Coca Cola as a contributing factor**. The root causes of NCDs is what you put on your plate, and with regards to the issue of obesity, the onus is on the individual to live an active lifestyle in order to prevent that from happening, and this is the very thing that the Coca Cola Games is trying to encourage, a healthier lifestyle. “Since **Coke Zero has zero calories, it can be useful in managing energy balance**. This is important in the management of body weight and therefore obesity and non-communicable diseases,” said Mr Tuitubou. (...) “If there are any corporate bodies that are willing to sponsor the same amount of money that Coca Cola Amatil is currently offering to the organisers, then they are most welcome to come forward but they should keep in mind the long term relationship that sponsoring the Fiji Secondary Schools Athletics competition means.” “If the corporate body is dedicated to supporting the development of sporting talent in the country through the secondary schools athletics competition on a long term basis and on the same level of sponsorship being offered by Coca Cola Amatil, I believe the organisers will be open to discussion,” said the Hon. Minister." |  | http://thestallionnewspaper.com/sports-minister-supports-coca-cola-sponsorship-for-secondary-school-athletics/ |
| A21 | FMF | Facebook | Constituency building | Seek involvement in the community | FMF Foods Ltd. March 8 at 2:44pm · We are here at Saru MGM Primary School for the International Women's Day Oratory Program !!! |  | https://www.facebook.com/FMFFoods |
| A22 | FMF | Facebook | Constituency building | Seek involvement in the community | FMF Foods Ltd. March 15 at 3:36pm · Our hearts and wishes go out to all our friends in Vanuatu who faced the brunt of tropical cyclone Pam over the weekend. | Picture of two men shaking hands in front of FMF boxes (Chow products - noodles) and a person with a "Chow" Tshirt Link to a news article: http://www.ifrc.org/en/news-and-media/news-stories/asia-pacific/vanuatu/vanuatu-braces-as-cyclone-pam-makes-landfall-68220/ | https://www.facebook.com/FMFFoods |
| A23 | FMF | Facebook | Constituency building | Seek involvement in the community | FMF Foods Ltd staff members contributed more than $30,000 worth of food to the Ministry of Rural and Maritime Development and National Disaster Management in Suva yesterday. The company's business development manager, Jim Taniela, said this was their contribution to relief efforts in Vanuatu following the devastation recently caused by Tropical Cyclone Pam. "FMF has advised our distributor in Port Vila, Vanuatu to start providing $7000 worth of FMF products to Red Cross when Tropical Cyclone Pam was still on category four," he said. The ministry's acting permanent secretary, Setareki Tale, acknowledged the efforts on behalf of Government. "This donation will supplement what Government has already collected from communities," he said. He said the ministry had goods worth about $230,000 that was ready to be shipped to Vanuatu with the complement of Pacific Agencies (Fiji) Ltd, a subsidiary of Swire Shipping. | Also posted on the FMF Facebook page | http://www.fijitimes.com/story.aspx?id=301451 |
| A24 | FMF | Facebook | Constituency building | Seek involvement in the community | Chow Games - Annual athletics meet for primary schools in Fiji organized by Fiji Primary Schools Athletic Association. |  | https://www.facebook.com/pages/Chow-Games/560604227340798 |
| A25 | FMF | News | Policy substitution | Policy substitution | The Fijian Government has established a partnership with the Fiji Food Industry Group to help combat and reduce Non communicable diseases in Fiji. While speaking at the World Consumer Rights Day yesterday, the Minister for Industry Trade and Tourism, Faiyaz Siddiq Koya addressed the initiatives that were going to be put in place. He said: “Some of the major food companies in Fiji have undertaken initiatives in voluntarily reducing the levels of sodium, fat and sugar.” These Companies are Nestle Trading Fiji Ltd, Coca-Cola Amatil, Motibhai Group, Food Processors Limited, Flour Mills of Fiji and McDonald’s. The Fiji Food Industry Group has holistically committed to a plan of action that ensures a co-ordinated and comprehensive approach towards this initiative. Specific guidelines are being introduced around marketing of food and beverages for children. |  | http://fijisun.com.fj/2015/03/25/healthy-living-is-priority-koya/ |
| A26 | FMF | News | Constituency building | Establish relationships with policy makers | The Fijian Government has established a partnership with the Fiji Food Industry Group to help combat and reduce Non communicable diseases in Fiji. These Companies are Nestle Trading Fiji Ltd, Coca-Cola Amatil, Motibhai Group, Food Processors Limited, Flour Mills of Fiji and McDonald’s. A joint working group has over seen the implementation of the action plans successfully with the food industry through consultations. There has been widespread adoption and acceptance of the initiatives undertaken by the industry. “The approach has also fostered a greater working relationship and closer ties with the Government to ensure effective collaboration is based on a partnership rather than the imposition of regulations, “This collaborative approach is a key to continuous and sustainable change in the environment for promotion of a healthier Fiji for a while providing consumers accessibility to healthy food,” explained Mr Koya. |  | http://fijisun.com.fj/2015/03/25/healthy-living-is-priority-koya/ |
| A27 | FMF | News | Information and messaging | Frame the debate on diet- and public health-related issues | The Fijian Government has established a partnership with the Fiji Food Industry Group to help combat and reduce Non communicable diseases in Fiji. These Companies are Nestle Trading Fiji Ltd, Coca-Cola Amatil, Motibhai Group, Food Processors Limited, Flour Mills of Fiji and McDonald’s. Companies who are part of the group are dedicated to proactively promote healthy active lifestyles and diets on the marketing side as well. Mr Koya highlight: “A key issue in Fiji is low intake of fruits and vegetables and high intake of starchy foods, most of which are not manufactured "Working on helping consumers change their eating patterns to a more holistic manner is the key to long-term success and integral to the efforts of the food industry." Specific guidelines are being introduced around marketing of food and beverages for children, this is so that providing children with a healthy dies is fully supported by parents. |  | http://fijisun.com.fj/2015/03/25/healthy-living-is-priority-koya/ |
| A28 | FMF | News | Constituency building | Seek involvement in the community | The FMF Foods Limited has made a donation of used clothes and food items worth more than $30,000 for the victims of Tropical Cyclone Pam in Vanuatu. While handing over the donation yesterday FMF Business Development Manager Jim Taniela says when cyclone Pam was on Category Four they had already advised their distributor in Port Villa to start gathering $7,000 worth of FMF products for those affected by the Cyclone Pam. Taniela said they will hand over the donation to the Ministry of Rural and Maritime Development and National Disaster Management who will then ship the donations to Vanuatu on the 15th of this month. |  | http://fijivillage.com/news/FMF-Foods-Ltd-makes-donation-over-30000-for-victims-of-TC-Pam-in-Vanuatu-sk59r2/ |
| A29 | FMF | Facebook | Constituency building | Seek involvement in the community | Round 3 of the 2015 Under-20 Chess Championship tournament leaders battling in long complex games |  | https://www.facebook.com/FMFFoods |
| A30 | Food Processors | News | Policy substitution | Policy substitution | The Fijian Government has established a partnership with the Fiji Food Industry Group to help combat and reduce Non communicable diseases in Fiji. While speaking at the World Consumer Rights Day yesterday, the Minister for Industry Trade and Tourism, Faiyaz Siddiq Koya addressed the initiatives that were going to be put in place. He said: “Some of the major food companies in Fiji have undertaken initiatives in voluntarily reducing the levels of sodium, fat and sugar.” These Companies are Nestle Trading Fiji Ltd, Coca-Cola Amatil, Motibhai Group, Food Processors Limited, Flour Mills of Fiji and McDonald’s. The Fiji Food Industry Group has holistically committed to a plan of action that ensures a co-ordinated and comprehensive approach towards this initiative. Specific guidelines are being introduced around marketing of food and beverages for children. |  | http://fijisun.com.fj/2015/03/25/healthy-living-is-priority-koya/ |
| A31 | Food Processors | News | Constituency building | Establish relationships with policy makers | The Fijian Government has established a partnership with the Fiji Food Industry Group to help combat and reduce Non communicable diseases in Fiji. These Companies are Nestle Trading Fiji Ltd, Coca-Cola Amatil, Motibhai Group, Food Processors Limited, Flour Mills of Fiji and McDonald’s. A joint working group has over seen the implementation of the action plans successfully with the food industry through consultations. There has been widespread adoption and acceptance of the initiatives undertaken by the industry. “The approach has also fostered a greater working relationship and closer ties with the Government to ensure effective collaboration is based on a partnership rather than the imposition of regulations, “This collaborative approach is a key to continuous and sustainable change in the environment for promotion of a healthier Fiji for a while providing consumers accessibility to healthy food,” explained Mr Koya. |  | http://fijisun.com.fj/2015/03/25/healthy-living-is-priority-koya/ |
| A32 | Food Processors | News | Information and messaging | Frame the debate on diet- and public health-related issues | The Fijian Government has established a partnership with the Fiji Food Industry Group to help combat and reduce Non communicable diseases in Fiji. These Companies are Nestle Trading Fiji Ltd, Coca-Cola Amatil, Motibhai Group, Food Processors Limited, Flour Mills of Fiji and McDonald’s. Companies who are part of the group are dedicated to proactively promote healthy active lifestyles and diets on the marketing side as well. Mr Koya highlight: “A key issue in Fiji is low intake of fruits and vegetables and high intake of starchy foods, most of which are not manufactured "Working on helping consumers change their eating patterns to a more holistic manner is the key to long-term success and integral to the efforts of the food industry." Specific guidelines are being introduced around marketing of food and beverages for children, this is so that providing children with a healthy dies is fully supported by parents. |  | http://fijisun.com.fj/2015/03/25/healthy-living-is-priority-koya/ |
| A33 | Goodman Fielder | News | Constituency building | Seek involvement in the community | The public has been watching on television ads, school children and teachers being encouraged to buy a particular brand of chicken in order to collect stickers that can be redeemed by the schools for educational products. Just lately, the same company is using the scheme to send teachers and children on exchange visits to New Zealand. Of course these educational rewards are good for the children and teachers. But why are school children, teachers and schools being used to foster the consumption of a particular brand of protein, whose inputs are largely imported? Similarly, student and teacher exchange would normally be a good exercise, to be funded by the Ministry of Education and tax-payers who could ensure appropriate representation, not managed by a private company to boost its chicken sales by selecting and rewarding those who buy more of its chicken. Note that while some may claim that it is merely one chicken brand competing against another chicken brand, I can assure you that not only will our consumers end up consuming more chicken, of all brands, but they will be consuming less of all our traditional proteins such as fresh fish and shell fish. Exactly similar processes are at work when noodles or rice companies compete through pervasive advertisements which increase the consumption of all noodles and rice, and decrease the consumption of traditional carbohydrates (as the facts clearly indicate). Why does the Ministry of Education allow such advertising schemes which use children’s need for educational products, to influence families decisions on what protein to buy or not to buy? |  | http://www.fijileaks.com/home/health-of-nation-under-threat-one-ministry-builds-and-another-destroys-worsening-nutrition-in-fiji-professor-wadan-narsey |
| A34 | Mc Donald's | Industry | Information and messaging | Frame the debate on diet- and public health-related issues | Can McDonald's food be part of a healthy, balanced diet?  Yes. **Many nutrition professionals agree that McDonald's food can be part of a healthy eating style** based on the s**ound nutrition principles of balance, variety and moderation**. One key to a healthy diet is to **moderate, not eliminate, favourite foods because eliminating foods is rarely successful in the long term**. It is important to eat a balanced diet over time, rather than focusing on any one food or meal. McDonald's **varied menu and range of serving sizes** make it easy to fit our food into a balanced diet and to create a range of meal combinations that fall within recommended guidelines for calories, fat and other nutrients. |  | http://www.mcdonaldsfiji.com/faq |
| A35 | Mc Donald's | Industry | Information and messaging | Frame the debate on diet- and public health-related issues | What's the meaning of your theme, "it's what i eat and what i do i'm lovin' it"?  The "it's what i eat and what i do" theme is part of **McDonald's ongoing commitment to inspire people to live balanced, active lifestyles**. The theme captures the message that **people should strive to find their own level of energy balance**. **Achieving energy balance means eating roughly the same number of calories that you use up throughout the day**. To put it simply, **Energy IN should be equal to Energy OUT.** Energy IN refers to the food that you eat every day, including meals, snacks and beverages. Energy OUT is the amount of energy you use when you walk, exercise, work and play and what your body uses for living (e.g., to keep your heart pumping, lungs breathing and blood circulating). When you maintain energy balance, your Energy IN is about the same as your Energy OUT. Your weight stays steady and your well-being improves. **When energy IN is greater than Energy OUT, weight is likely to be gained. Conversely, when Energy IN is less than Energy OUT, weight loss may be the result.** |  | http://www.mcdonaldsfiji.com/faq |
| A36 | Mc Donald's | News | Policy substitution | Policy substitution | The Fijian Government has established a partnership with the Fiji Food Industry Group to help combat and reduce Non communicable diseases in Fiji. While speaking at the World Consumer Rights Day yesterday, the Minister for Industry Trade and Tourism, Faiyaz Siddiq Koya addressed the initiatives that were going to be put in place. He said: “Some of the major food companies in Fiji have undertaken initiatives in voluntarily reducing the levels of sodium, fat and sugar.” These Companies are Nestle Trading Fiji Ltd, Coca-Cola Amatil, Motibhai Group, Food Processors Limited, Flour Mills of Fiji and McDonald’s. The Fiji Food Industry Group has holistically committed to a plan of action that ensures a co-ordinated and comprehensive approach towards this initiative. Specific guidelines are being introduced around marketing of food and beverages for children. |  | http://fijisun.com.fj/2015/03/25/healthy-living-is-priority-koya/ |
| A37 | Mc Donald's | News | Constituency building | Establish relationships with policy makers | The Fijian Government has established a partnership with the Fiji Food Industry Group to help combat and reduce Non communicable diseases in Fiji. These Companies are Nestle Trading Fiji Ltd, Coca-Cola Amatil, Motibhai Group, Food Processors Limited, Flour Mills of Fiji and McDonald’s. A joint working group has over seen the implementation of the action plans successfully with the food industry through consultations. There has been widespread adoption and acceptance of the initiatives undertaken by the industry. “The approach has also fostered a greater working relationship and closer ties with the Government to ensure effective collaboration is based on a partnership rather than the imposition of regulations, “This collaborative approach is a key to continuous and sustainable change in the environment for promotion of a healthier Fiji for a while providing consumers accessibility to healthy food,” explained Mr Koya. |  | http://fijisun.com.fj/2015/03/25/healthy-living-is-priority-koya/ |
| A38 | Mc Donald's | News | Information and messaging | Frame the debate on diet- and public health-related issues | The Fijian Government has established a partnership with the Fiji Food Industry Group to help combat and reduce Non communicable diseases in Fiji. These Companies are Nestle Trading Fiji Ltd, Coca-Cola Amatil, Motibhai Group, Food Processors Limited, Flour Mills of Fiji and McDonald’s. Companies who are part of the group are dedicated to proactively promote healthy active lifestyles and diets on the marketing side as well. Mr Koya highlight: “A key issue in Fiji is low intake of fruits and vegetables and high intake of starchy foods, most of which are not manufactured "Working on helping consumers change their eating patterns to a more holistic manner is the key to long-term success and integral to the efforts of the food industry." Specific guidelines are being introduced around marketing of food and beverages for children, this is so that providing children with a healthy dies is fully supported by parents. |  | http://fijisun.com.fj/2015/03/25/healthy-living-is-priority-koya/ |
| A39 | Mc Donald's | News | Constituency building | Seek involvement in the community | Give them Love, Give them Hope, Give them a reason to Smile was the catchphrase used by a group of companies in Nadi last Saturday to help fulfil the wishes of three children suffering from cancer. The children are between the ages of three and 17 and are admitted at the Lautoka Hospital. The companies made their wishes come true. Yees Cold Storage Seafood Limited initiated the help and supported by Unilever – the world’s biggest ice cream manufacturer (Streets Ice cream), Novotel Hotel Nadi, Sofitel Fiji Resort and Spa, McDonald’s Fiji Nadi outlet, Fiji Sun and Vodafone Fiji. (...) “McDonald’s came on board with free lunch and face painting. Afterwards, the children were taken down to Yee’s Headquarters at Namaka where the presentation was made. |  | http://fijisun.com.fj/2015/04/20/gifts-for-nadi-cancer-children/ |
| A40 | Motibhai | News | Policy substitution | Policy substitution | The Fijian Government has established a partnership with the Fiji Food Industry Group to help combat and reduce Non communicable diseases in Fiji. While speaking at the World Consumer Rights Day yesterday, the Minister for Industry Trade and Tourism, Faiyaz Siddiq Koya addressed the initiatives that were going to be put in place. He said: “Some of the major food companies in Fiji have undertaken initiatives in voluntarily reducing the levels of sodium, fat and sugar.” These Companies are Nestle Trading Fiji Ltd, Coca-Cola Amatil, Motibhai Group, Food Processors Limited, Flour Mills of Fiji and McDonald’s. The Fiji Food Industry Group has holistically committed to a plan of action that ensures a co-ordinated and comprehensive approach towards this initiative. Specific guidelines are being introduced around marketing of food and beverages for children. |  | http://fijisun.com.fj/2015/03/25/healthy-living-is-priority-koya/ |
| A41 | Motibhai | News | Constituency building | Establish relationships with policy makers | The Fijian Government has established a partnership with the Fiji Food Industry Group to help combat and reduce Non communicable diseases in Fiji. These Companies are Nestle Trading Fiji Ltd, Coca-Cola Amatil, Motibhai Group, Food Processors Limited, Flour Mills of Fiji and McDonald’s. A joint working group has over seen the implementation of the action plans successfully with the food industry through consultations. There has been widespread adoption and acceptance of the initiatives undertaken by the industry. “The approach has also fostered a greater working relationship and closer ties with the Government to ensure effective collaboration is based on a partnership rather than the imposition of regulations, “This collaborative approach is a key to continuous and sustainable change in the environment for promotion of a healthier Fiji for a while providing consumers accessibility to healthy food,” explained Mr Koya. |  | http://fijisun.com.fj/2015/03/25/healthy-living-is-priority-koya/ |
| A42 | Motibhai | News | Information and messaging | Frame the debate on diet- and public health-related issues | The Fijian Government has established a partnership with the Fiji Food Industry Group to help combat and reduce Non communicable diseases in Fiji. These Companies are Nestle Trading Fiji Ltd, Coca-Cola Amatil, Motibhai Group, Food Processors Limited, Flour Mills of Fiji and McDonald’s. Companies who are part of the group are dedicated to proactively promote healthy active lifestyles and diets on the marketing side as well. Mr Koya highlight: “A key issue in Fiji is low intake of fruits and vegetables and high intake of starchy foods, most of which are not manufactured "Working on helping consumers change their eating patterns to a more holistic manner is the key to long-term success and integral to the efforts of the food industry." Specific guidelines are being introduced around marketing of food and beverages for children, this is so that providing children with a healthy dies is fully supported by parents. |  | http://fijisun.com.fj/2015/03/25/healthy-living-is-priority-koya/ |
| A43 | Natural Waters | News | Constituency building | Seek involvement in the community | The Ministry of Infrastructure and Transport has received two shipping containers of bottled water, worth $50,000, donated by Natural Waters of Viti Ltd, bottlers of Fiji Water. The aid is for relief efforts for schools and health centres in parts of the country affected by the prolonged dry period. |  | http://fijisun.com.fj/2014/10/08/bottled-water-for-schools/ |
| A44 | Nestle | News | Policy substitution | Policy substitution | The Fijian Government has established a partnership with the Fiji Food Industry Group to help combat and reduce Non communicable diseases in Fiji. While speaking at the World Consumer Rights Day yesterday, the Minister for Industry Trade and Tourism, Faiyaz Siddiq Koya addressed the initiatives that were going to be put in place. He said: “Some of the major food companies in Fiji have undertaken initiatives in voluntarily reducing the levels of sodium, fat and sugar.” These Companies are Nestle Trading Fiji Ltd, Coca-Cola Amatil, Motibhai Group, Food Processors Limited, Flour Mills of Fiji and McDonald’s. The Fiji Food Industry Group has holistically committed to a plan of action that ensures a co-ordinated and comprehensive approach towards this initiative. Specific guidelines are being introduced around marketing of food and beverages for children. |  | http://fijisun.com.fj/2015/03/25/healthy-living-is-priority-koya/ |
| A45 | Nestle | News | Constituency building | Establish relationships with policy makers | The Fijian Government has established a partnership with the Fiji Food Industry Group to help combat and reduce Non communicable diseases in Fiji. These Companies are Nestle Trading Fiji Ltd, Coca-Cola Amatil, Motibhai Group, Food Processors Limited, Flour Mills of Fiji and McDonald’s. A joint working group has over seen the implementation of the action plans successfully with the food industry through consultations. There has been widespread adoption and acceptance of the initiatives undertaken by the industry. “The approach has also fostered a greater working relationship and closer ties with the Government to ensure effective collaboration is based on a partnership rather than the imposition of regulations, “This collaborative approach is a key to continuous and sustainable change in the environment for promotion of a healthier Fiji for a while providing consumers accessibility to healthy food,” explained Mr Koya. |  | http://fijisun.com.fj/2015/03/25/healthy-living-is-priority-koya/ |
| A46 | Nestle | News | Information and messaging | Frame the debate on diet- and public health-related issues | The Fijian Government has established a partnership with the Fiji Food Industry Group to help combat and reduce Non communicable diseases in Fiji. These Companies are Nestle Trading Fiji Ltd, Coca-Cola Amatil, Motibhai Group, Food Processors Limited, Flour Mills of Fiji and McDonald’s. Companies who are part of the group are dedicated to proactively promote healthy active lifestyles and diets on the marketing side as well. Mr Koya highlight: “A key issue in Fiji is low intake of fruits and vegetables and high intake of starchy foods, most of which are not manufactured "Working on helping consumers change their eating patterns to a more holistic manner is the key to long-term success and integral to the efforts of the food industry." Specific guidelines are being introduced around marketing of food and beverages for children, this is so that providing children with a healthy dies is fully supported by parents. |  | http://fijisun.com.fj/2015/03/25/healthy-living-is-priority-koya/ |
| A47 | New World IGA | Facebook | Information and messaging | the evidence base on diet and public health-related issues | NEWWORLD IGA February 23 at 8:50pm · Check out Newworld IGA's **wide variety of organic products, now making it easier for you to live a healthy lifestyle**! Available now at all Newworld IGA Stores. |  | https://www.facebook.com/pages/NEWWORLD-IGA/252868404886324 |
| A48 | Tappoo | Industry | Constituency building | Seek involvement in the community | in conjunction with the official opening of TappooCity, we are equally pleased to announce today a **$21,000 donation through the Ministry of Education, for the purposes of funding Educational Expenses for needy school students at Primary, Secondary and Tertiary School levels in the Nadroga Province.**  This donation closely follows our recent **$12,000 contribution to the Sigatoka Hospital Maternity Ward, and our $10,000 contribution to the Save the Children Fund** - both of which were announced at the Official Opening of our new Bottling Plant in Lautoka. |  | http://www.tappoo.com.fj/index.php/84-chairmansmessage |
| A49 | Tappoo | Industry | Constituency building | Seek involvement in the community | **TappooCity is proudly sponsoring the 2015 National Blitz Chess Championship** that will be held at FASANOC Olympic House on Saturday 31 January from 10:00 AM. “We are extremely proud to be able to extend our support for Chess in developing the minds of youth and young people of Fiji”, says Tappoo Group Director Remal Tappoo. “**Tappoo has a unique history of assisting various organizations. For many decades, Tappoo has taken great pride in sponsoring various sporting organizations at club, district, provincial and even national levels in Fiji**”.  “TappooCity has generously sponsored all prizes and up to 50 consolation prizes for participants,” states Acting-President Calvin Prasad |  | http://www.tappoo.com.fj/index.php/chess-championship |
| A50 | Tappoo | Industry | Constituency building | Seek involvement in the community | THE Tappoo family today paid tribute to Fiji’s special father of special children Frank Hilton, for his invaluable commitment and gifted, the Hilton Organization, $5,000 to assist it in its efforts. |  | http://www.tappoo.com.fj/index.php/news/80-news/344 |
| A51 | Tappoo | Industry | Constituency building | Seek involvement in the community | TAPPOO Fiji Limited has branched out to do charity work in education for the Nadroga and Navosa district. The company presented a cheque for $10,000 to help 13 secondary schools in the district. And the assistance would benefit 250 students. Minister for Education Filipe Bole commended the support from the company and said it would assist students reach the highest level of secondary education |  | http://www.tappoo.com.fj/index.php/news/80-news/306-tappoo-assists-13-schools |
| A52 | Unilever | News | Constituency building | Seek involvement in the community | Give them Love, Give them Hope, Give them a reason to Smile was the catchphrase used by a group of companies in Nadi last Saturday to help fulfil the wishes of three children suffering from cancer. The children are between the ages of three and 17 and are admitted at the Lautoka Hospital. The companies made their wishes come true. Yees Cold Storage Seafood Limited initiated the help and supported by Unilever – the world’s biggest ice cream manufacturer (Streets Ice cream), Novotel Hotel Nadi, Sofitel Fiji Resort and Spa, McDonald’s Fiji Nadi outlet, Fiji Sun and Vodafone Fiji. (...) Unilever helped through the sale of Pink Magnum ice cream where part of the sale proceeds went towards helping children with cancer. |  | http://fijisun.com.fj/2015/04/20/gifts-for-nadi-cancer-children/ |
